# Type of Primary Nb_5_Si_3_ and Precipitation of Nb_ss_ in αNb_5_Si_3_ in a Nb-8.3Ti-21.1Si-5.4Mo-4W-0.7Hf (at.%) Near Eutectic Nb-Silicide-Based Alloy

**DOI:** 10.3390/ma11060967

**Published:** 2018-06-07

**Authors:** Conor McCaughey, Panos Tsakiropoulos

**Affiliations:** Department of Materials Science and Engineering, The University of Sheffield, Hadfield Building, Mappin Street, Sheffield S1 3JD, UK; conormccaughey1987@gmail.com

**Keywords:** Nb-silicide-based alloys, phase equilibria, solidification, intermetallics

## Abstract

The Nb-silicide-based alloy of near eutectic composition (at.%) Nb-21.1Si-8.3Ti-5.4Mo-4W-0.7Hf (alloy CM1) was studied in the cast and heat-treated (1500 °C/100 h) conditions. The alloy was produced in the form of buttons and bars using three different methods, namely arc-melting, arc-melting and suction casting, and optical floating zone (OFZ) melting. In the former two cases the alloy solidified in water-cooled copper crucibles. Buttons and suction-cast bars of different size, respectively of 10 g and 600 g weight and 6 mm and 8 mm diameter, were produced. The OFZ bars were grown at three different growth rates of 12, 60 and 150 mm/h. It was confirmed that the type of Nb_5_Si_3_ formed in the cast microstructures depended on the solidification conditions. The primary phase in the alloy CM1 was the βNb_5_Si_3_. The transformation of βNb_5_Si_3_ to αNb_5_Si_3_ had occurred in the as cast large size button and the OFZ bars grown at the three different growth rates, and after the heat treatment of the small size button and the suction-cast bars of the alloy. This transformation was accompanied by subgrain formation in Nb_5_Si_3_ and the precipitation of Nb_ss_ in the large size as cast button and only by the precipitation of Nb_ss_ in the cast OFZ bars. Subgrains and precipitation of Nb_ss_ in αNb_5_Si_3_ was observed in the small size button and suction-cast bars after the heat treatment. Subgrains formed in αNb_5_Si_3_ after the heat treatment of the OFZ bars. The partitioning of solutes and in particular of Mo and Ti was key to this phase transformation. Subgrain formation was not necessary for precipitation of Nb_ss_ in αNb_5_Si_3_, but the partitioning of solutes was essential for this precipitation.

## 1. Introduction

Environmental and performance targets for future aeroengines could be met with aerofoil (blade) materials having capabilities beyond those of Ni-based superalloys [1,2,3]. Refractory metal intermetallic composites (RMICs) based on the Nb-Si system that are known as Nb-silicide-based alloys or Nb silicide in situ composites can offer a balance of properties and can meet some of the property goals. The microstructures of these new alloys often contain only the most desirable phases, namely the bcc Nb solid solution (Nb_ss_) and tetragonal Nb_5_Si_3_ silicide, but other intermetallics also can be present, for example the tetragonal Nb_3_Si silicide and the C14-NbCr_2_ Laves and A15-Nb_3_X (X = Al, Ge, Si, Sn) phases [3,4,5,6,7]. To date, the great majority of the reported research is about cast and heat-treated alloys (for example, see [3,6,7]). There is also some research on alloys prepared using powder metallurgy (PM) [8] and on alloys processed using spark plasma sintering (SPS) [9].

Most of the studied alloys have been prepared as small (<20 g) buttons. Few studies have used larger size buttons and ingots [10]. The great majority of studies used arc-melting with non-consumable tungsten electrode. Plasma melting has been used in a few studies. A very limited number of alloys have been prepared using directional solidification [11]. Optical floating zone (OFZ) melting also has been used as a “directional” solidification technique [12]. OFZ uses focused light to melt the material that moves through the stationary hot zone resulting to containerless solidification. In OFZ, growth at a constant rate is possible but the temperature gradient in the melt cannot be kept constant.

In the Nb-Si binary [13] the βNb_5_Si_3_ compound (tI32, prototype W_5_Si_3_, D8_m_) melts congruently at T_m_ = 2535 °C and is stable between this temperature and about 1920 °C, depending on the phase diagram. The αNb_5_Si_3_ compound (tI32, prototype Cr_5_B_3_, D8_l_) is stable below about 1920 °C to room temperature. In Nb-rich hypereutectic Nb-Si binary alloys the βNb_5_Si_3_ is the primary phase that forms from the melt for Si concentrations greater than some value that could be from 17 to 21.1 at.%, depending on the phase diagram [14]. The αNb_5_Si_3_ can form via the eutectoid Nb_3_Si → Nb + αNb_5_Si_3_ and βNb_5_Si_3_ → αNb_5_Si_3_ + NbSi2 or peritectoid βNb_5_Si_3_ + Nb_3_Si → αNb_5_Si_3_ reactions. Contamination by interstitials promotes the formation of metastable γNb_5_Si_3_ compound (hP16, prototype Mn_5_Si_3_, D8_8_) in binary Nb-Si alloys [13]. The eutectic L → Nb + Nb_3_Si can be suppressed under Rapid Solidification conditions and replaced by the metastable eutectic L → Nb + βNb_5_Si_3_. The liquid composition of the former eutectic has been reported to be in the range 15.3 to 18.7 at.% in published Nb-Si binary phase diagrams [14]. In the Ti-Si binary [15] the hexagonal Ti_5_Si_3_ silicide (hP16, prototype Mn_5_Si_3_, D8_8_) melts congruently at 2130 °C and is stable to room temperature. (In the literature, the Nb_3_Si, Nb_5_Si_3_, Ti_5_Si_3_ are referred to as compounds, intermetallics, phases or silicides [3,8,13,14]).

Titanium is an important addition in Nb-silicide-based alloys. It improves their oxidation resistance, reduces their density and improves the toughness of the Nb_ss_ [16,17]. Titanium substitutes Nb in βNb_5_Si_3_ and αNb_5_Si_3_3 [18]. This substitution affects the stability and changes the properties of the alloyed (Nb,Ti)_5_Si_3_ silicides [19]. At least 7 versions of the Nb-Ti-Si liquidus projection have been reported [20,21,22,23,24,25,26]. Some of the projections were based solely on experimental data and others were calculated. In some projections the type of (Nb,Ti)_5_Si_3_ was not specified, others indicated that only the β(Nb,Ti)_5_Si_3_ could form from the melt and others that the β(Nb,Ti)_5_Si_3_ or the α(Nb,Ti)_5_Si_3_ could form from the melt depending on alloy composition. The presence of α(Nb,Ti)_5_Si_3_ areas in the liquidus projection indicates that Ti can stabilize the low temperature αNb_5_Si_3_ to high temperatures.

Alloyed tetragonal βNb_5_Si_3_ and/or tetragonal αNb_5_Si_3_ and/or hexagonal γNb5Si3 have been reported in the microstructures of Nb-silicide-based alloys [5,6,18,27,28,29]. The properties of alloyed Nb_5_Si_3_ depend on its crystal structure. First-principles calculations of physical properties, Cauchy pressures, Pugh’s index of ductility and Poisson ratio showed that as the Ti concentration in (Nb,Ti)_5_Si_3_ increased (a) the bulk moduli of the α(Nb,Ti)_5_Si_3_, β(Nb,Ti)_5_Si_3_ and γ(Nb,Ti)_5_Si_3_ silicides decreased, (b) the shear and elastic moduli increased for the α(Nb,Ti)_5_Si_3_ and γ(Nb,Ti)_5_Si_3_ silicides and decreased for β(Nb,Ti)_5_Si_3_, (c) the α(Nb,Ti)_5_Si_3_ and γ(Nb,Ti)_5_Si_3_ silicides became less ductile and the β(Nb,Ti)_5_Si_3_ became more ductile and (d) the linear thermal expansion coefficients of the α(Nb,Ti)_5_Si_3_ and β(Nb,Ti)_5_Si_3_ silicides decreased, and the anisotropy of the coefficient of thermal expansion did not change significantly [19].

The type of primary Nb_5_Si_3_ in an Nb-silicide-based alloy defines the path along which the micro-structure develops during solidification and subsequent processing. Also, it is important for the properties of the alloy. Is it possible for the stability of the low temperature tetragonal αNb_5_Si_3_ to be extended to the melting temperature with alloying? For a given alloy, could the primary βNb_5_Si_3_ transform to αNb_5_Si_3_ during the solidification of large size buttons and ingots? Recently, it was shown that the βNb_5_Si_3_ to αNb_5_Si_3_ transition temperature decreased significantly with increasing Ti concentration in (Nb,Ti)_5_Si_3_ and that the hexagonal γ(Nb,Ti)_5_Si_3_ became stable only at Ti concentrations above approximately 50 at.% Ti [19].

The motivation for the research presented in this paper was to investigate the microstructure of a near eutectic Nb-silicide-based alloy with Ti, Hf and refractory metal additions that was selected based on the results of a recent study [30], in particular whether the type of primary silicide depended on solidification conditions.

## 2. Experimental

The Nb-silicide-based alloy of near eutectic composition Nb-21.1Si-8.3Ti-5.4Mo-4W-0.7Hf (at.%) (alloy CM1) was selected for this study. The choice of the nominal composition of this alloy was based on [30]. The objectives of the research reported in this paper were to find out (a) whether the type of primary Nb_5_Si_3_ depended on the solidification conditions and (b) whether the size (weight) of the button could have an effect on the transformation of Nb_5_Si_3_ during solidification. Objective (a) was addressed by casting the alloy using three different methods, namely arc-melting, arc-melting and suction casting, and OFZ processing. In the former two cases the alloy solidified in water-cooled copper crucibles. For objective (b) it was decided to use buttons of different sizes but typical of those used in previous and current research on Nb-silicide-based alloys, namely buttons of 10 g and 600 g in weight.

The alloy was prepared using high purity elements (purity better than 99.99 wt.%). The elemental charge was arc-melted five times before solidification. Bars of 6 mm and 8 mm diameter were prepared using arc-melting and suction casting. OFZ bars were grown at Hokkaido University in Japan using a mirror furnace with four xenon lamps, counter-rotation of the support and feeder rods at 30 rpm and pulling of the system downwards at three different velocities (see below) in an Ar atmosphere [31]. Under normal gravity a short melt zone is desirable owing to its greater stability compared with long zones (see Section 4.1 below). OFZ processing aimed to keep the ratio of the length to the diameter of the melt zone equal to 1. The floating zone moved through the solid by moving the solid rods (feeder and grown solid) downwards (meaning the movement of the S/L interface was in the opposite direction).

Cylindrical bars 80 mm long and 10 mm diameter were cut from 600 g arc-melted buttons and grown using OFZ with growth rates of 12, 60 and 150 mm/h. These growth rates were significantly lower than those in arc melting. Indeed, in the OFZ facility used, the growth rate of 4200 mm/h (not employed in this research) is comparable with that in arc melting [31].

The arc melted alloy with the two different weights is referred to below as CM1-10g and CM1-600g, and the suction-cast alloy with the two different diameter bars is referred to as CM1-6mm and CM1-8mm, respectively. The OFZ alloy is referred to as CM1-OFZ.

Specimens of the alloy CM1 prepared using the aforementioned methods were heat treated at 1500 °C for 100 h as described in [18]. The cast and heat-treated microstructures were characterized using powder X-ray diffraction (XRD) and scanning electron microscopy (SEM) with energy dispersive X-ray spectroscopy (EDS).

The microstructures were studied using back scatter electron (BSE) imaging in Inspect F, JEOL 6400, Camscan Mk2 and Philips 500 and XL30 scanning electron microscopes (SEMs). Quantitative chemical analyses of the microstructures and phases were done using EDS in JOEL 6400, Camscan Mk2 and Philips 500 SEMs that were equipped with high purity well-polished elemental standards. These gave the actual chemical composition of the alloy and the phases and eutectic in its microstructure. Qualitative X-ray maps were produced on a Philips XL30 FEG SEM using a Bruker AXS XFLASH Detector 4010 and Bruker Espirit EDS analysis software. All imaging and EDS analyses were done with an accelerating voltage of 20 kV. The analysis data given in the paper includes the average and standard deviation. In addition to these values, the minimum and maximum analysis values are given for CM1-OFZ.

Powder samples were used for X-ray diffraction (XRD) with monochromatic copper radiation in a STOE machine with 2θ range of 20 to 120 degrees and step size of 0.05 degrees. The phases were determined using WINX^POW^ software and ICDD PDF-4+ 2012 database packages. Powder XRD can be affected by the introduction of plastic strain in the samples. Powders of Nb-silicide-based alloys are not significantly deformed during their preparation [32].

## 3. Results

### 3.1. Cast Alloy

**Figure 1:**
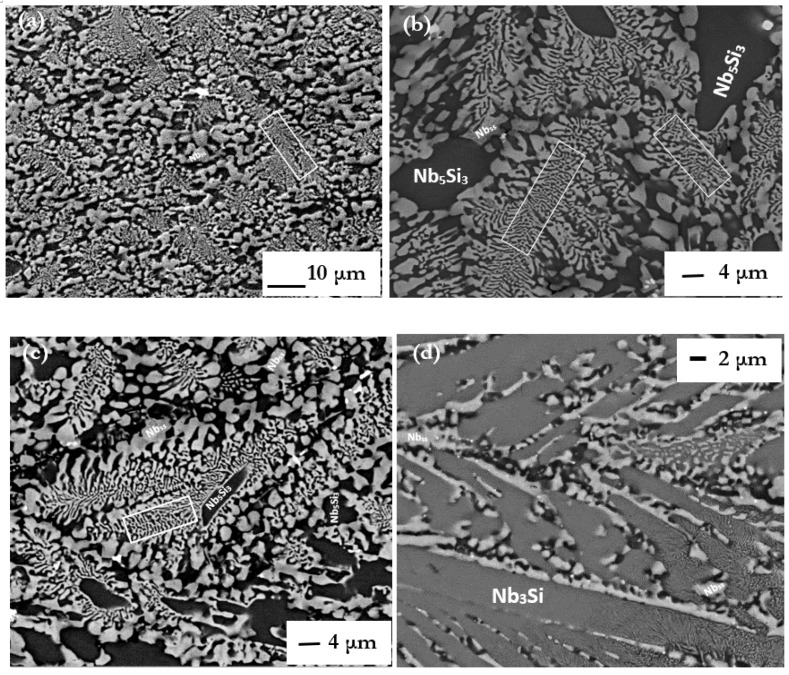

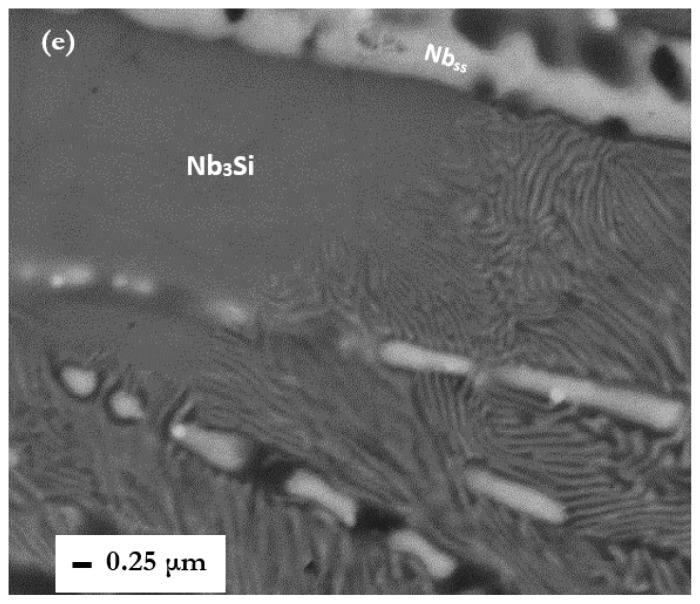
As cast alloy CM1 (**a**) CM1-10g button, (**b**) CM1-6mm (bulk) and (**c**–**e**) CM1-8mm suctions cast bars, (**c**) bulk, (**d**) and (**e**) from area of high cooling rate and 18.6 < Si < 19.2 at.%. Rectangles show lamellar microstructures. The lamellar microstructure resulting from the eutectoid transformation of Nb3Si is shown in the right hand side of (**e**).

**Figure 2:**
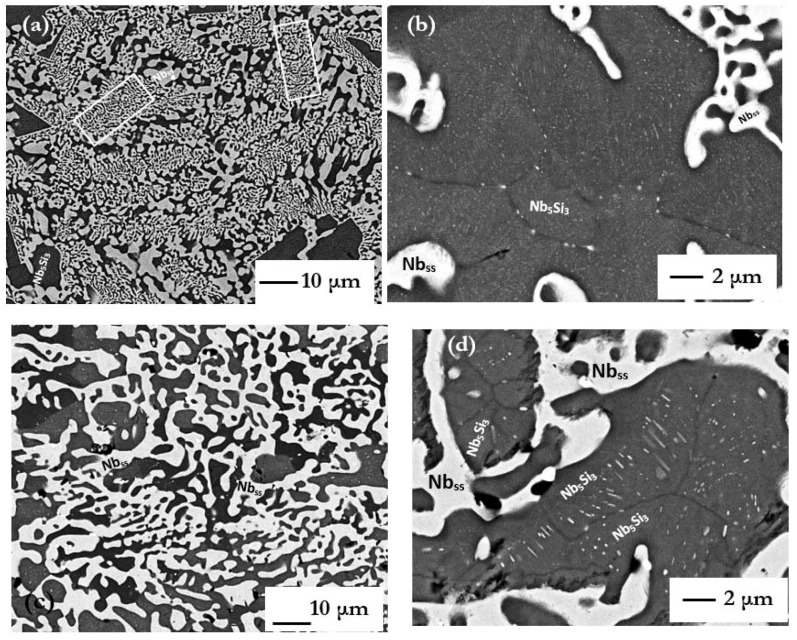
Alloy CM1-600g button (**a**) and (**b**) as cast, (**c**) and (**d**) heat treated. The rectangles show lamellar microstructure. For (**b**) and (**d**) see text.

**Figure 3:**
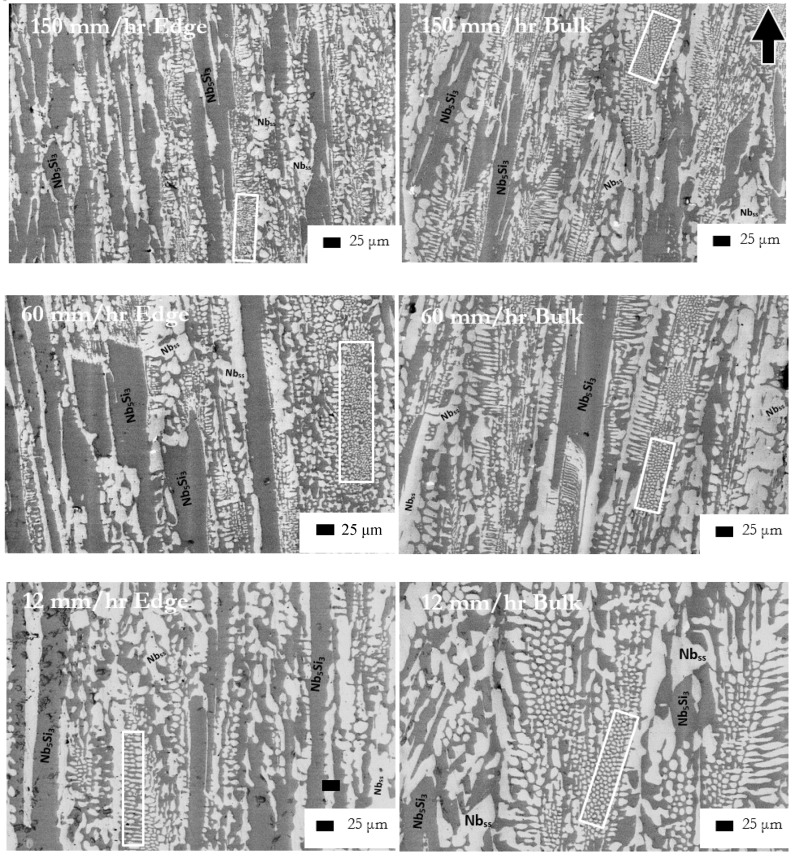
BSE images of CM1-OFZ processed at 150 mm/h, 60 mm/h and 12 mm/h, respectively from top to bottom. Left hand images show the microstructure at the edge of the bar and right hand images show the bulk microstructure. The arrow indicates the growth direction. Rectangles show lamellar microstructure.

The microstructures (i) of the arc melted CM1-10g and CM1-600g buttons are shown respectively in Figure 1a and Figure 2a, (ii) of the arc melted and suction-cast CM1-6mm and CM1-8mm bars are shown respectively in Figure 1b–e, and (iii) of the CM1-OFZ bars grown at the three different growth rates of 12, 60 and 150 mm/h are shown in Figure 3. The XRD data for (i), (ii) and (iii) is shown respectively in Figures S1–S3 in supplemental data. The XRD data for the type of Nb_5_Si_3_ in (i) to (iii) is summarized in Table 1. In the latter, the type of silicide confirmed by XRD is indicated by X. In CM1-10g, CM1-600g, CM1-6mm, CM1-8mm and CM1-OFZ there was a very low volume fraction of hafnia (HfO_2_) that was not detected by XRD.

The average composition (at.%) of CM1-10g, CM1-600g, CM1-6mm and CM1-8mm respectively was (60 ± 0.3)Nb-(22.6 ± 0.35)Si-(8.8 ± 0.1)Ti-(4.7 ± 0.1)Mo-3.1W-0.8Hf, (60.9 ± 0.3)Nb-(22.7 ± 0.6)Si-(8.5 ± 0.2)Ti-(5.2 ± 0.3)Mo-2.1W-0.7Hf, (58 ± 0.6)Nb-(23.4 ± 0.5)Si-(9.7 ± 0.3Ti)-(5.4 ± 0.3)Mo-(2.9 ± 0.2)W-0.6Hf and (57.5 ± 0.4)Nb-(21.8 ± 0.5)Si-(9.2 ± 0.5)Ti-(7.6 ± 0.2)Mo-3.2W-0.7Hf. All actual compositions were very close to the nominal one. There was no macrosegregation of Si and other elements.

The starting bars for the OFZ solidification processing of the alloy CM1 were cut from the 600 g button with the average composition given above. The analysis data for the three different growth rates is given in Table 2. In Table 2 large area analysis refers to quantitative analysis of the alloy microstructure at low magnification (X350) and lamellar microstructure analysis refers to quantitative analysis only of the lamellar microstructure (see Figure 1, Figure 2 and Figure 3). The average composition of the bulk and the outer part (shown as edge in Table 2) of the grown bar was different at each growth rate and between growth rates. As the growth rate increased the edge became richer in Si, and the difference in Ti concentration between the bulk and edge decreased. The surfaces of the OFZ grown cylindrical bars were not absolutely smooth.

The microstructure of the alloy CM1 in the arc melted buttons, arc melted and suction-cast bars and OFZ grown bars consisted of Nb_5_Si_3_ and a lamellar microstructure of the Nb_ss_ and Nb_5_Si_3_ phases, see Figure 1, Figure 2 and Figure 3. In the CM1-10g, CM1-6mm and CM1-8mm the βNb_5_Si_3_ was observed (Figures S1 and S2 in supplemental data) but in the CM1-600g and CM1-OFZ the αNb_5_Si_3_ was observed (Figures S1 and S3 in supplemental data), see also Table 1. The average composition of the Nb_5_Si_3_ and Nb_ss_ phases was essentially the same for the arc melted and arc melted and suction-cast alloy.

The average composition (at.%) of Nb_5_Si_3_ in CM1-10g, CM1-600g, CM1-6mm and CM1-8mm respectively was (52.8 ± 0.6)Nb-(37.4 ± 0.5)Si-(6.6 ± 0.8)Ti-(2.5 ± 0.3)Mo-0.5W-0.2Hf, (54.3 ± 0.1)Nb-(37.1 ± 0.4)Si-(6.1 ± 0.2)Ti-(2 ± 0.2)Mo-0.1W-0.3Hf, (51.3 ± 0.2)Nb-(37.4 ± 0.4)Si-(7 ± 0.1)Ti-(3.5 ± 0.3)Mo-0.6W-0.3Hf, and (51.9 ± 0.4)Nb-(37 ± 0.4)Si-(6.4 ± 0.1)Ti-(3.7 ± 0.2)Mo-0.7W-0.4Hf. In the 5-3 silicide the Nb was substituted by Hf, Mo, Ti and W and the concentrations of Hf and W in the Nb_5_Si_3_ were very low.

The average composition (at.%) of Nb_ss_ in CM1-10g, CM1-600g, CM1-6mm and CM1-8mm respectively was (74.7 ± 0.6)Nb-(1.6 ± 0.6)Si-(8 ± 1.3)Ti-(9.6 ± 0.5)Mo-(6.4 ± 1.4)W-0.4Hf, (73.5 ± 0.7)Nb-(1.7 ± 0.9)Si-(9.4 ± 0.6)Ti-(10 ± 0.4)Mo-(4.6 ± 1.1)W-0.7Hf, (72.3 ± 0.6)Nb-(1.2 ± 0.5)Si-(7.5 ± 0.3)Ti-(11.1 ± 0.3)Mo-(7.7 ± 1)W-0.2Hf, (73.7 ± 0.7)Nb-(1.6 ± 0.9)Si-(6.9 ± 0.5)Ti-(9.9 ± 0.4)Mo-(7.8 ± 0.7)W-0.1Hf. The solid solution was richer in Mo and W compared with the nominal alloy composition and the Si concentration agreed with previous work on Nb-silicide-based alloys [6,28,33].

The average composition (at.%) of the lamellar microstructure of Nb_ss_ and Nb_5_Si_3_ in CM1-10g, CM1-600g, CM1-6mm and CM1-8mm respectively was (60.7 ± 0.2)Nb-(19.9 ± 0.8)Si-(9.5 ± 0.2)Ti-(6 ± 0.6)Mo-(3.4 ± 0.2)W-0.5Hf, (62.5 ± 0.8)Nb-(19.4 ± 0.8)Si-(8.8 ± 0.4)Ti-(5.9 ± 0.2)Mo-(2.5 ± 0.3)W-0.8Hf, (55.8 ± 0.3)Nb-(22.5 ± 0.3)Si-((10 ± 0.2)Ti-((8 ± 0.3)Mo-2.9W-0.7Hf and (58.3 ± 0.5)Nb-(20.4 ± 1.1)Si-(9.4 ± 0.1)Ti-(8 ± 0.4)Mo-(3.3 ± 0.2)W-0.7Hf. In CM1-6mm, the average Si concentration was higher, about 22.5 at.%, and the concentration of Hf, Mo, Ti and W were not significantly different from those in CM1-8mm.

In CM1-10g the solid solution grains that were rich in Ti and Hf were poor in Mo and W and vice versa. The relationship between the W and Ti concentrations in the Nb_ss_ is given in Figure S4a in supplemental data, which shows that “Ti and W did not like each other in the Nb_ss_”, see also [34]. The concentrations of the refractory metals in the Nb_ss_ in CM1-600g were related to those of Si and Ti. When the Si concentration in the Nb_ss_ was higher than 1 at.% the concentration of W was lower than 4.2 at.% and when the concentration of Si was lower than 1 at.% the W concentration was higher than 6.3 at.%. The lowest concentrations of Ti (<9 at.%) corresponded to the highest Mo and W concentrations and vice versa. In CM1-OFZ, in the microstructure in the outer parts (edge) of the bars grown at the three different growth rates the Nb_ss_ was richer in W and Mo, particularly W, and richer in Ti in the bulk (Table 2). There was significant variation in the concentrations of Ti and W in the solid solution; as the concentration of Ti increased that of W decreased (Figure S4b in supplemental data).

The chemical compositions of Nb_5_Si_3_ in the bulk and edge of the CM1-OFZ bars were essentially the same (Table 2). The Nb_ss_ and Nb_5_Si_3_ lamellar microstructure was richer in Si and Ti in the bulk, and in Mo and W in the edge of the bars (Table 2).

In CM1-OFZ the microstructure was similar for all growth rates. Large grains of Nb_5_Si_3_ that were elongated in the direction of growth were typically surrounded by solid solution. Between the silicide grains, areas of a lamellar microstructure of Nb_ss_ and Nb_5_Si_3_ formed. These areas also were elongated in the direction of growth and were surrounded by a coarser more randomly orientated lamellar microstructure. The morphology of the microstructure did not change with growth rate.

Comparison of the bulk and edge microstructures for all growth rates showed several similarities, most obvious of which was the increased number of large silicide grains at the edge compared with the bulk and the reduced volume fraction of the lamellar microstructure. Variations in the Si concentration in the large area and lamellar microstructure analyses were persistent at all growth rates (note the larger standard deviation values in Table 2). This was especially evident for the 12 and 60 mm/h growth rates. For example, for the growth rate of 12 mm/h the Si concentration varied by about 2 at.% or more in all analyses. For the highest growth rate (150 mm/h) the variation in Si concentration was observed only at the edge of the bar.

Evidence for a very small volume fraction of Nb_3_Si and for the eutectoid Nb_3_Si → Nb_ss_ + αNb_5_Si_3_ reaction was found only in one area of the bottom of CM1-6mm that was poorer in Si than the average composition (Figure 1d,e). The Si concentration in Nb_3_Si was 24.7 at.%. This microstructure was observed in the part of the bar that had solidified under high cooling rate. The above eutectoid reaction “was frozen in” and thus the eutectoid microstructure was observed. Detailed study of the cast CM-10g, CM1-600g, CM1-6mm and CM1-OFZ did not find any evidence for Nb_3_Si and the above eutectoid reaction.

**Figure 4:**
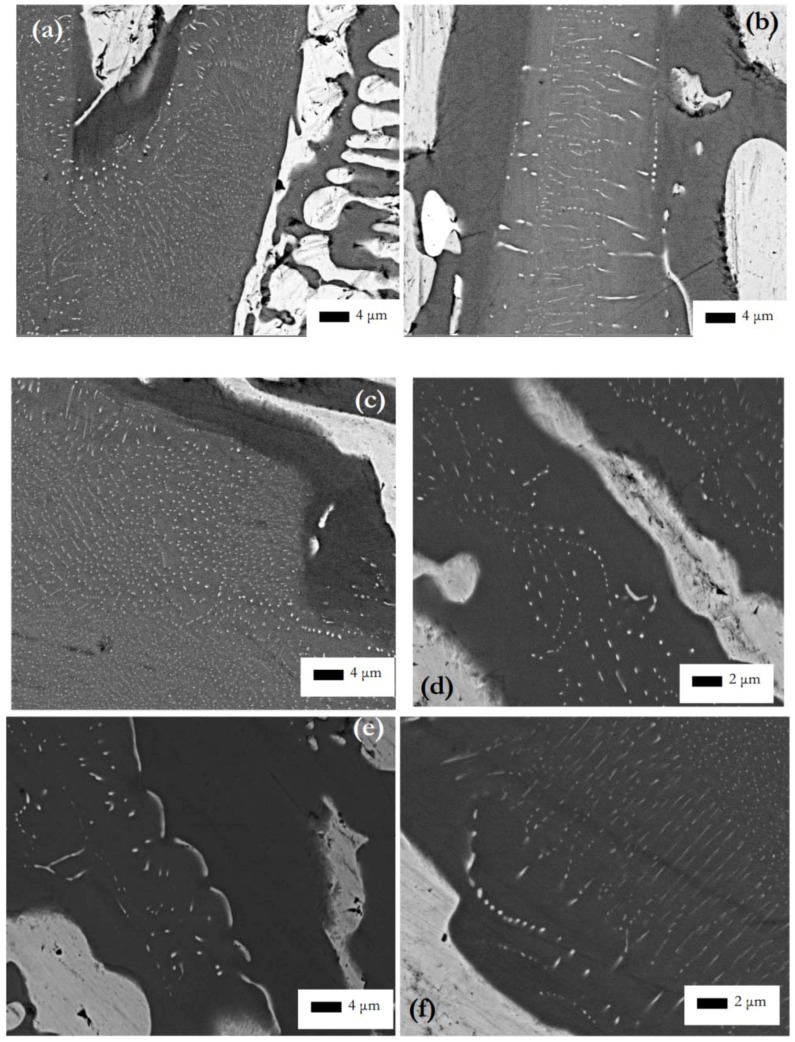
Backscattered electron images showing regions of light contrast, including precipitates, within the silicide phase in CM1-OFZ for the growth rates of (**a**) 150, (**b**) 60, (**c**) 12 and (**d**) 150, (**e**,**f**) 12 mm/h.

In CM1-600g in the Nb_5_Si_3_ grains there was evidence of subgrains and of fine precipitates of a second phase with same contrast under BSE imaging conditions as the Nb_ss_, see Figure 2b. In CM1-OFZ and for all three growth rates there was no evidence of subgrain formation in Nb_5_Si_3_ but there was evidence of fine precipitates of a second phase in Nb_5_Si_3_ grains. These fine precipitates exhibited the same contrast under BSE imaging conditions as the Nb_ss_, see Figure 4. These fine precipitates in Nb_5_Si_3_ were detectable only under BSE imaging in the FEG-SEMs Inspect F and Philips XL30.

Figure 4a–c shows the internal microstructure in silicide grains of CM1-OFZ grown at 150, 60 and 12 mm/h. These images clearly show two distinct regions within the silicide; a lighter contrast area with light contrast precipitates and a darker contrast area located closer to the solid solution phase with no precipitates. The regions with precipitates were usually found in the larger silicide grains but sometimes also were found in the silicide in the lamellar microstructure, see Figure 4a. The precipitates seemed to form with two distinct morphologies at all growth rates; spherical and elongated. Both morphologies were found in the same silicide grains suggesting that the precipitates grew perpendicular or parallel or at an angle to the section plane. Also, there was evidence of thin, long and straight or curved lamellae of precipitates, or of fine precipitates forming a “curve” in the microstructures, and evidence for “break-up” of the long thin lamellae. The curvature of the fine precipitates seemed to follow that of the Nb_ss_/Nb_5_Si_3_ interface opposite. X-ray elemental maps of a Nb_5_Si_3_ grain with fine precipitates are shown in Figure S5 in supplemental data. The maps showed higher concentrations of Nb and Mo in the lighter contrast areas of the silicide and higher concentrations of Ti in the darker areas. Tungsten seemed to show a slight affinity for the lighter contrast area of the silicide. However, due to the very low concentration of W in Nb_5_Si_3_ (Table 2) the “segregation” of W (if any) is not certain.

The microstructures that were observed in the cast alloy CM1 are summarized in Table S1 in supplemental data.

### 3.2. Heat-Treated Alloy

**Figure 5:**
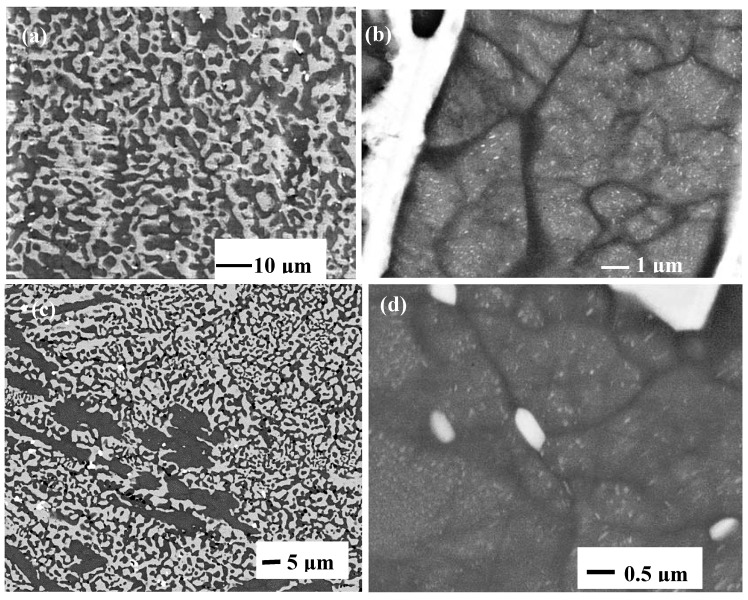

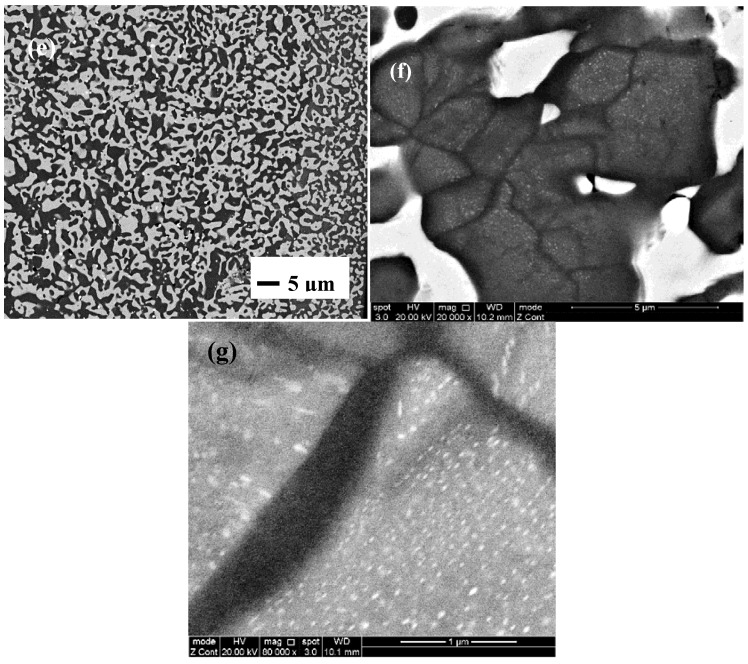
Heat-treated alloy CM1 (**a**), (**b**) and (**g**) 10 g button, (**c**) and (**d**) 6 mm diameter suction-cast bar, (**e**) and (**f**) 8 mm diameter suction-cast bar, (**c**) to (**f**) images from bulk of bars.

The microstructures of the heat-treated CM1-10g, CM1-600g, CM1-6mm, CM1-8mm and CM1-OFZ consisted of Nb_ss_ and αNb_5_Si_3_ (Figures S1, S2 and S6 in supplemental data and Table 1) and a very low volume fraction of HfO_2_ particles. The microstructure of CM1-OFZ was very similar to the cast one. All the microstructures had coarsened (Figure 2c, Figure 3 and Figure 5a,c,e). The coarsening was more noticeable in CM1-10g, CM1-600g, CM1-6mm and CM1-8mm.

The average composition of the heat-treated specimens was the same as that of the cast buttons, suction-cast bars and the OFZ bars. In the latter, the composition of Nb_5_Si_3_ was essentially the same as in the cast bars for all three growth rates. The noticeable changes in the chemical composition of the Nb_ss_ were as follows: (i) the concentration of Si was reduced to below 0.7 at.% (ii) there were some Nb_ss_ grains where no Si was analyzed (in other word Nb_ss_ with no Si was formed [34,35]) and (iii) the concentration of Mo had decreased by about 2 at.%. The chemical compositions of the lamellar microstructures were similar for the three growth rates and essentially very close to those of the OFZ cast bars, with the only exception that the Mo concentration had decreased by about 1 at.%.

In CM1-600g and CM1-8mm the αNb_5_Si_3_ exhibited darker and brighter contrasts owing to its different Ti content. For example, in CM1-600g the Ti rich silicide had 14.7 < Ti < 15.6 at.% compared with the “normal” silicide, which had 6.1 < Ti < 8 at.%, and in the Nb_ss_ the Ti concentration had decreased compared with the cast alloy.

**Figure 6:**
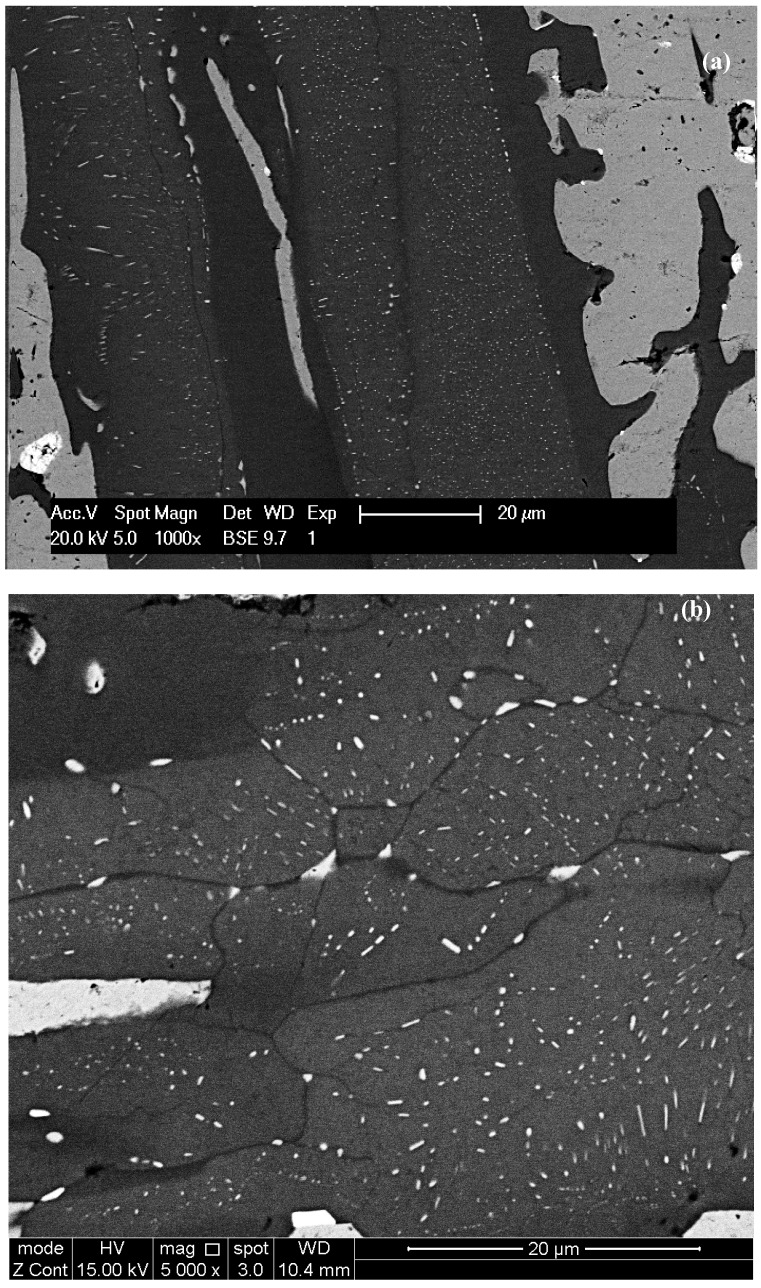
BSE images of heat-treated CM1-OFZ showing precipitation of Nb_ss_ and sub-grains in Nb_5_Si_3_ (**a**) 60 mm/h, (**b**) 150 mm/h.

In the Nb_5_Si_3_ grains in the heat-treated CM1-10g, CM1-6mm and CM1-8mm there was evidence of subgrains and fine precipitates of a second phase with same contrast under BSE imaging conditions as the Nb_ss_, see Figure 5b,d,f. Some of the precipitates were elongated (e.g., see Figure 2d) and might had formed with an orientation relationship in Nb_5_Si_3_ (Figure 5g). In the CM1-6mm the size of the precipitates was finer than those in CM1-10g but similar to those in the cast CM1-600g. The subgrains that were observed in the Nb_5_Si_3_ grains in the cast CM1-600g were still present and the second phase particles had coarsened (Figure 2d). Subgrain formation in Nb_5_Si_3_ grains also was observed in CM1-OFZ bars grown at the three different growth rates. Typical microstructures are shown in Figure 6, where it should be noted that second phase particles were formed in the light contrast parts of αNb_5_Si_3_, as was the case with the cast CM1-OFZ.

The microstructures that were observed in the heat-treated alloy CM1 are summarized in Table S1 in supplemental data.

## 4. Discussion

### 4.1. Cast Alloy

The alloy of this research exhibited a variety of microstructures, most of which comprised the Nb_ss_ and Nb_5_Si_3_, the latter being either the high temperature βNb_5_Si_3_ or the low temperature αNb_5_Si_3_. The Nb_3_Si and the fine products of its eutectoid decomposition were also observed in CM1-8mm, in an area where the suction-cast bar had experienced high cooling rate during solidification. Characteristic features of eutectics and/or eutectoids were observed in lamellar microstructures. The latter were formed around dendrites of Nb_5_Si_3_, and in most cases Nb_ss_ haloes were formed in-between Nb_5_Si_3_ grains and lamellar microstructure.

The discussion will consider first the eutectic between Nb_ss_ and βNb_5_Si_3_ (metastable eutectic in the Nb-Si binary). This was observed in the cast CM1-10g and CM1-6mm (Figure 1a,b), where only the presence of βNb_5_Si_3_ was confirmed by XRD (Figures S1 and S2 in supplemental data), and in CM1-8mm where the XRD confirmed only the βNb_5_Si_3_ (Figure 1c and Figure S2 in supplemental data). However, in an area of CM1-8mm that had solidified under high cooling rates there was evidence for Nb_3_Si and its eutectoid transformation to Nb_ss_ and αNb_5_Si_3_ (Figure 1d,e) (the case for CM1-600g and CM1-OFZ will be discussed below separately). The Si concentration in CM1-10g, CM1-6mm and CM1-8mm (respectively 22.6, 23.4 and 21.8 at.%) was above the eutectic composition in the Nb-Si binary (reported to be in the range 15.3 to 18.7 at.% [14]) and the Si concentration of the eutectic was in the range of values for the metastable Nb_ss_ and βNb_5_Si_3_ eutectic in the binary [30].

In eutectic solidification the coupled zone gives the range of compositions and growth temperatures for which the coupled eutectic morphology leads the growth front. The Nb-Si binary has eutectic coupled zones skewed towards the silicides [36]. The coupled zone depends on the relative change of the interface (growth) temperature of different morphological forms (eutectic, dendrites) as a function of composition and growth rate. Under a set of given growth conditions, the growth form (eutectic, dendrite) with the highest interface temperature will lead the growth front.

**Figure 7:**
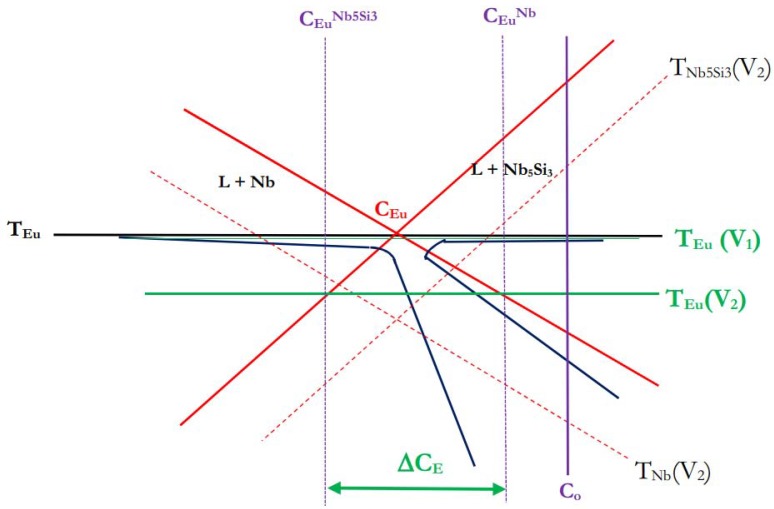
Schematic diagram of the skewed coupled zone of the Nb + βNb_5_Si_3_ metastable eutectic. Red continuous lines show the liquidus of Nb and βNb_5_Si_3_ and dashed red lines the growth temperatures T_i_ (i = Nb, βNb_5_Si_3_) dendrites at growth rate V_2_. The black horizontal line is the eutectic temperature T_Eu_. Drawing based on [37].

Figure 7 shows a schematic diagram of the skewed eutectic zone for the metastable eutectic in the Nb-Si binary, based on [37]. From constitutional supercooling theory the maximum velocity of the solid-liquid interface V_S/L_ for coupled Nb + βNb_5_Si_3_ eutectic will be V_S/L_^CS^ = G_L_ D_L_ [m_βNb5Si3_ (C_o_ − C_Eu_)] where m_βNb5Si3_ is the slope of the liquidus of βNb_5_Si_3_. When V_S/L_ exceeds V_S/L_^CS^ (V_2_ > V_S/L_^CS^ in Figure 7), for an alloy of composition C_o_ the growth temperature of the βNb_5_Si_3_ is above that of T_Eu(V2)_. The liquid composition between the primary βNb_5_Si_3_ and the coupled eutectic S/L interface will be in the range ∆C_E_ = C_Eu_^Nb^ − C_Eu_^Nb5Si3^. At a low growth rate V_1_ < V_S/L_^CS^ the growth temperature T_Eu(V1)_ of the coupled Nb + βNb_5_Si_3_ eutectic can be higher than the growth temperatures of each phase, namely T_Nb(V1)_ and T_Nb5Si3(V1)_ (note that the growth temperatures T_i_ (i = Nb, βNb_5_Si_3_) at the growth rate V_1_ are not shown in Figure 7) and thus according to the criterion of maximum growth temperature the alloy of composition C_o_ will solidify as coupled Nb + βNb_5_Si_3_ eutectic.

The microstructures seen in CM1-10g, CM1-6mm and CM1-8mm that formed at different growth rates (growth temperatures) under conditions of positive temperature gradient G_L_ in the melt (G_L_ > 0) can be explained with the help of Figure 7. As the solidification conditions changed coupled Nb + βNb_5_Si_3_ eutectic with/out primary βNb_5_Si_3_ formed.

Next, the formation of Nb_3_Si in CM1-8mm will be considered. There was no macrosegregation in CM1-8mm. Its Si concentration was lower than that of CM1-6mm and close to the liquid concentration (C_Lp_) of the L + βNb_5_Si_3_ → Nb_3_Si peritectic in the Nb-Si binary, for which the highest reported value is 21.1 at.% Si [14]. It is highly likely that the melt of CM1-8mm was either to the left (primary phase Nb_3_Si) or to the right (primary phase βNb_5_Si_3_) of C_Lp_.

With Nb_3_Si as the primary phase (alloy composition C_o_ < C_Lp_) the solidification path in the Nb-Si binary is L → L + (Nb_3_Si)_primary_ → (Nb_3_Si)_primary_ + (Nb + Nb_3_Si)_eutectic_. In this case, no βNb_5_Si_3_ is formed. If all the Nb_3_Si transforms, there will be the fine mixture of eutectoid Nb and αNb_5_Si_3_, if not, there will also be some remnant of Nb_3_Si. The evidence in the bottom of CM1-8mm (Figure 1d,e) is for the latter.

The data in Table 1 and Table S1 in supplemental data would suggest that in CM1-600g and CM1-OFZ at all three growth rates the αNb_5_Si_3_ was the product of the βNb_5_Si_3_ → αNb_5_Si_3_ transformation that occurred under slow cooling and that the lamellar microstructures in the large button and OFZ bars were indeed prior Nb_ss_ + βNb_5_Si_3_ eutectics (formed as discussed above for CM1-10g, CM1-6mm, and CM1-8mm, see Figure 7) in which the βNb_5_Si_3_ silicide had transformed to αNb_5_Si_3_. Considering current knowledge of the Nb-Si binary, there is no metastable eutectic between Nb_ss_ and αNb_5_Si_3_. The fact that the Nb_3_Si and its eutectoid transformation were observed in the area of high cooling rate in CM1-8mm is attributed to the solidification path of the melt in that area (see above). The high cooling rate allowed us to “catch” the formation of Nb_3_Si and its early stages of eutectoid decomposition during solid state cooling.

To summarize, in the alloy CM1 the primary phase was the βNb_5_Si_3_, the Nb_ss_ + βNb_5_Si_3_ eutectic formed and in the large button (i.e., CM1-600 g) and CM1-OFZ, both of which cooled slower compared with the CM1-10g, CM1-6mm and CM1-8mm, the βNb_5_Si_3_ transformed to αNb_5_Si_3_ during solid state cooling. Please note that if only the large button or OFZ bars grown at each of the three growth rates of this study had been investigated, the conclusion that αNb_5_Si_3_ was the primary phase would be misleading.

The microstructures in CM1-600g and CM1-OFZ were similar to those observed in the cast CM1-10g (Figure 1a and Figure 2a) but the XRD data of the former two confirmed the presence of Nb_ss_ and αNb_5_Si_3_ (Figures S1 and S3 in supplemental data). The other difference between the large and small buttons (i.e., CM1-600g and CM1-10g) was the subgrain formation with fine precipitates of a second phase exhibiting same contrast as the Nb_ss_ in the large button, Figure 2b. The difference between CM1-600g and CM1-OFZ was that in the latter no subgrains were observed in the αNb_5_Si_3_ grains with fine precipitates. In CM1-10g, CM1-6mm and CM1-8mm the βNb_5_Si_3_ had no subgrains and no fine second phase precipitates of a phase with contrast similar to that of the Nb_ss_, but subgrains and fine precipitates formed in αNb_5_Si_3_ in CM1-10g, CM1-6mm and CM1-8mm after the heat treatment (Figure 5). Table S1 in supplemental data summarizes the phases observed in the cast alloy CM1.

The subgrain formation in CM1-600g is related to the βNb_5_Si_3_ to αNb_5_Si_3_ transformation in the cast CM1-600g and could be attributed to “recovery” of the 5-3 silicide during solid state cooling driven by the strain energy arising from differences in thermal contraction between Nb_ss_ and Nb_5_Si_3_. First, subgrain formation in Nb_5_Si_3_ will be considered using the Nb-Si binary (i.e., ignoring for the time being the role of the other solutes).

First-principles calculations performed in our group showed that the Young’s moduli of Nb, βNb_5_Si_3_ and αNb_5_Si_3_ respectively are 105, 269 and 291 GPa [38]. The CTE (coefficient of thermal expansion) of Nb is 7.3 to 7.6 10^−6^ K^−1^ depending on purity. The βNb_5_Si_3_ and αNb_5_Si_3_ exhibit anisotropy in CTE and the ratio of CTE values α_a_/α_c_ along the a and c axes of their lattices (i.e., the CTE anisotropy ratio) is different for each phase, namely 0.783 and 0.658 for αNb_5_Si_3_ and βNb_5_Si_3_, respectively [19,39]. The CTE values of βNb_5_Si_3_ and αNb_5_Si_3_ along their a-axes are very close (8.961 10^−6^ K^−1^ and 8.777 10^−6^ K^−1^) but those along their c-axes are different, namely 11.095 10^−6^ K^−1^ and 13.331 10^−6^ K^−1^, respectively [19]. All the aforementioned values are for the unalloyed phases and are expected to change as they become alloyed (see below and [39]).

Allowing for the strain energy in Nb_5_Si_3_ to arise from the CTE anisotropy, meaning the lower the α_a_/α_c_ ratio the higher the anisotropy, the subgrain formation as the βNb_5_Si_3_ transformed to αNb_5_Si_3_ would be accompanied by a reduction in strain energy (the “driving force”), the latter driving “recovery” and subgrain formation in the silicide.

In CM1-OFZ and for all growth rates the αNb_5_Si_3_ was present in the cast microstructures (Figure S3 in supplemental data) but no subgrains were observed in the 5-3 silicide grains. Given that thermal stresses in the “grown” solid can arise during solidification (see below) and that the sign and magnitude of these stresses depends on changes in cross section during OFZ and transport phenomena (heat and mass transfer) in the melt zone (see below), the absence of subgrains in CM1-OFZ was attributed to reduced strain energy due to the thermal stresses arising during OFZ counter-balancing stresses arising from the anisotropy of CTE of the 5-3 silicide (see below).

Subgrain formation was not observed in CM1-10g, CM1-6mm and CM1-8mm owing to the faster cooling during solid state cooling which did not give enough time for the transformation of the βNb_5_Si_3_ to αNb_5_Si_3_ and for “recovery” processes to occur. To our knowledge, subgrain formation in Nb_5_Si_3_ in binary Nb-Si alloys has not been reported in the literature.

Next, we consider the results of this research in the context of the available data in the literature about Nb-Si-Mo, Nb-Si-W, Nb-Ti-Si and Mo-Si-W ternary phase equilibria and the properties of α(Nb,Ti)_5_Si_3_ and β(Nb,Ti)_5_Si_3_ (i.e., alloyed 5-3 silicide). Below, alloy compositions are given in at.%.

The Nb_3_Si was destabilized by small Mo additions in Nb-Si-Mo alloys [40]. In Nb-16Si-xMo alloys with x ≤ 2 the primary phase was the Nb_ss_ and for x > 2 the β(Nb,Mo)_5_Si_3_ + Nb_ss_ eutectic replaced the (Nb,Mo)_3_Si + Nb_ss_ eutectic. In Nb-19Si-xMo alloys the primary phase was the (Nb,Mo)_3_Si for x ≤ 2 and the β(Nb,Mo)_5_Si_3_ for x > 2. In the latter alloys only the β(Nb,Mo)_5_Si_3_ + Nb_ss_ eutectic was stable for x ≥ 4 but both the β(Nb,Mo)_5_Si_3_ + Nb_ss_ and (Nb,Mo)_3_Si + Nb_ss_ eutectics were formed for x = 3. In Nb-20Si-xMo alloys the primary phase was the β(Nb,Mo)_5_Si_3_, and peritectic (Nb,Mo)_3_Si and (Nb,Mo)_3_Si + Nb_ss_ eutectic formed for x = 1 and only β(Nb,Mo)_5_Si_3_ + Nb_ss_ eutectic for x ≥ 3 [41].

The composition of βNb_5_Si_3_ in the alloy Nb-19Si-3Mo was Nb-38.6Si-0.8Mo according to Ma et al. [41]. Sekido et al. [42] reported higher Mo solubilities in Nb_5_Si_3_, namely 3.6 at.% and 5.2 at.% in αNb_5_Si_3_ and βNb_5_Si_3_, respectively. Solubilities of 2.5 at.% Mo and 0.6 at.% Mo in βNb_5_Si_3_ and αNb_5_Si_3_ were reported respectively for the cast and heat-treated conditions of the alloy Nb-18Si-5Al-5Cr-5Mo and slightly lower Mo concentration in the βNb_5_Si_3_ in the cast alloy Nb-24Ti-18Si-5Al-5Cr-5Mo [43]. In other words, the data in the literature shows that the solubility of Mo is different in βNb_5_Si_3_ and αNb_5_Si_3_, depends on the presence or not of Ti in the alloy and also on the concentration of Mo in the alloy.

Sekido et al. [42] reported that Mo stabilized the βNb_5_Si_3_ and that the transformation βNb_5_Si_3_ → αNb_5_Si_3_ (i) did not occur when the concentration of Mo in Nb_5_Si_3_ exceeded 6 at.% and (ii) depended on the cooling rate. After heat treatment at 1400 °C for 100 h a mixture of both βNb_5_Si_3_ and αNb_5_Si_3_ was observed when the Mo concentration in Nb_5_Si_3_ was 4 or 5 at.%. Also, they reported that a two phase Nb_ss_ + αNb_5_Si_3_ or Nb_ss_ + βNb_5_Si_3_ phase equilibria could be attained in the Nb-Si-Mo ternary at 1700 °C.

In the Nb-Si-W ternary phase equilibria the W has the same effect as Mo, namely it destabilizes the Nb_3_Si and stabilizes the Nb_ss_ and βNb_5_Si_3_ eutectic when the W concentration is above ≈ 3 at.% [40]. Addition of 10 at.% W allowed formation of amorphous Nb-Si-W alloys [44], which would suggest that W enhances the undercool-ability of Nb-Si-W alloys. Continuous solid solutions between βNb_5_Si_3_ and W_5_Si_3_ have been reported in cast ternary alloys [45] where the transformation of βNb_5_Si_3_ to αNb_5_Si_3_ did not allow the continuous solid solutions to persist to lower temperatures. The W solubility in αNb_5_Si_3_ was about 1 at.% [40]. Mo and W form continuous solid solutions and Mo_5_Si_3_ and W_5_Si_3_ (and βNb_5_Si_3_) have the same prototype.

The early data that supported the construction of the Nb-Ti-Si liquidus projection(s) did not identify the structure of 5-3 silicides in the studied alloys (i.e., did not clarify which Nb_5_Si_3_ polymorph was formed) and the liquidus projection gave a Nb_5_Si_3_ area, without specifying whether this was the βNb_5_Si_3_ or the αNb_5_Si_3_ or the hexagonal γNb_5_Si_3_ [21]. Geng et al. [23] proposed a liquidus projection with a large αNb_5_Si_3_ area. Li et al. [25] revised the Nb-Ti-Si liquidus projection and proposed only a very narrow area for the αNb_5_Si_3_ in the center of the projection. A small area of αNb_5_Si_3_ in the center of the projection has also been proposed by Gigolotti et al. [26]. The Scheil solidification path for Nb-19Si-5Hf was given as L → L + Nb(Hf)_3_Si → (Nb,Hf)_3_Si + (Nb,Hf,Si)_ss_ [46].

Sekido et al. [42] studied arc melted Nb-xMo-36Si and Nb-xMo-37.5Si (at.%, x = 0 to 10) alloys in the as cast and heat-treated conditions. They reported that Nb_ss_ precipitates formed in both βNb_5_Si_3_ and αNb_5_Si_3_ after heat treatment at 1300 °C for 20 h. Precipitation of Nb_ss_ in βNb_5_Si_3_ was not observed in the cast condition but after heat treatment at 1500 °C for 100 h. Plate shaped Nb_ss_ precipitates were formed in βNb_5_Si_3_ and the orientation relationship {$\bar{1}$01}_Nb_ //{2$\bar{1}$0}_βNb5Si3_, <111> // <121>_βNb5Si3_ was observed. In the αNb_5_Si_3_ that formed from the βNb_5_Si_3_ → αNb_5_Si_3_ transformation after heat treatment at 1500 °C for 100 h there was Nb_ss_ precipitation in αNb_5_Si_3_ and for these precipitates two orientation relationships were observed, namely (01$\bar{1}$)_Nbss_ // (12$\bar{3}$)_αNb5Si3_, [133]_Nbss_ // [111]_αNb5Si3_ and ($\bar{1}$12)_Nbss_ // (1$\bar{1}$0)_αNb5Si3_, [110]_Nbss_ // [110]_αNb5Si3_, which agreed with the orientation relationships reported for eutectoid Nb_ss_/αNb_5_Si_3_ lamellae by Sekido et al. [47] and Miura et al. [48]. Sekido et al. [42] suggested that the βNb_5_Si_3_ → αNb_5_Si_3_ transformation occurred before the Nb_ss_ precipitated or simultaneously and attributed the precipitation of Nb_ss_ to the βNb_5_Si_3_ exhibiting temperature dependent solubility owing to anti-site substitution of Nb and Mo atoms for Si sites. The off-stoichiometry towards the Si rich side of the phase diagram was suggested to be attained by substitutional, i.e., anti-site defects and not by vacancies on Nb sites.

In the alloy CM1, the formation of primary βNb_5_Si_3_ is consistent with the Nb-Si-Mo and Nb-Si-W liquidus projections. In the Nb-Ti-Si liquidus projection by Li et al. [25] the primary phase in the alloy CM1 is the βNb_5_Si_3_ when Nb, Mo, Hf and W are considered as equivalent, meaning the alloy CM1 is considered to be an (Nb,Mo,Hf,W)-Ti-Si alloy. The suppression of Nb_3_Si and the formation of the Nb_ss_ + βNb_5_Si_3_ eutectic is also consistent with the liquidus projections for the Nb-Si-Mo and Nb-Si-W ternaries. The solidification of the Nb_ss_ + βNb_5_Si_3_ eutectic can be explained with the help of Figure 7. The formation of Nb_3_Si in CM1-8mm can be explained as discussed above but also can be related to variations in Si and Mo concentration of the melt and the data from [41] for Nb-19Si-xMo and Nb-20Si-xMo alloys. The presence of αNb_5_Si_3_ in CM1-600g can be attributed to the βNb_5_Si_3_ → αNb_5_Si_3_ transformation during solid state cooling that depends on the Mo concentration in the βNb_5_Si_3_ and on cooling rate. Phase equilibria data for 1700 °C for Nb-Si-Mo [40] and Nb-Si-W [41] shows that the αNb_5_Si_3_ is in equilibrium with Nb_ss_ (in the Nb-Si binary the αNb_5_Si_3_ is stable below about 1920 °C). The observation of αNb_5_Si_3_ only in the cast CM1-600g and not in the cast CM1-10g, CM1-6mm or CM1-8mm would suggest that the slower cooling of the larger ingot was enough for the βNb_5_Si_3_ → αNb_5_Si_3_ transformation to occur, consistent with the observations of Sekido et al. [42].

Subgrains were observed in the αNb_5_Si_3_ in CM1-600g. First-principles calculations have been performed in our group to show the effect of Ti substituting Nb in the βNb_5_Si_3_ and αNb_5_Si_3_ on the moduli of elasticity of β(Nb,Ti)_5_Si_3_ and α(Nb,Ti)_5_Si_3_ and their CTE values [19]. The Young’s modulus (E) of αNb_5_Si_3_ increased with Ti substituting Nb but that of βNb_5_Si_3_ decreased. For 12.5 at.% Ti in each of the two silicides the E values were 314 and 244 GPa, respectively. The CTE of the βNb_5_Si_3_ and αNb_5_Si_3_ also changed and continued to exhibit anisotropy and the ratio α_a_/α_c_ (i.e., the CTE anisotropy ratio) was different for each phase, namely 0.797 and 0.611 for αNb_5_Si_3_ and βNb_5_Si_3_, respectively, when 12.5 at.% Ti substituted Nb in Nb_5_Si_3_. The CTE values of βNb_5_Si_3_ and αNb_5_Si_3_ along their c-axes were very close (10.682 10^−6^ K^−1^ and 10.980 10^−6^ K^−1^) but those along their a-axes were different, namely 8.510 10^−6^ K^−1^ and 6.709 10^−6^ K^−1^. In other words, the substitution of Nb by Ti in the tetragonal Nb_5_Si_3_ makes the low temperature αNb_5_Si_3_ polymorph more isotropic (the ratio of CTE values α_a_/α_c_ increases) and the high temperature βNb_5_Si_3_ polymorph more anisotropic.

Considering strain energy in alloyed βNb_5_Si_3_ to arise from the CTE anisotropy, meaning the lower the α_a_/α_c_ ratio the higher the anisotropy, the subgrain formation as the β(Nb,Ti)_5_Si_3_ transformed to α(Nb,Ti)_5_Si_3_ would be accompanied by a reduction in strain energy (the “driving force” for recovery and subgrain formation).

The above discussion has ignored the role of Mo substituting Nb atoms in Nb_5_Si_3_ because no data is available, but pointed to some special role played by Ti and Mo in the alloy CM1 (owing to the formation of subgrains and fine Nb_ss_ precipitates in αNb_5_Si_3_ in CM1-600g). We shall expand on this point below where the case for CM1-OFZ will be considered.

#### OFZ

The floating zone process is containerless and surface tension forces “hold” the melt. The upper limit of the length of the melt zone (meaning the length beyond which the zone becomes unstable and collapses) is proportional to (γ_LV_/ρg)^1/2^ where γ_LV_ is the surface tension of the melt, ρ is melt density and g is the gravitational acceleration [49]. For elemental Si, Nb, Mo the (γ_LV_/ρg)^1/2^ is large (respectively 0.17, 0.16 and 0.15) which means that necking in the melt zone is not severe. For Nb silicide-based alloys with densities in the range 6.5 < ρ < 7.5 g/cm^3^ [28,29,33,50,51] this ratio is in the range 0.167 to 0.18 (not accounting for changes in γ_LV_).

In gravity conditions, convection in the liquid can arise from surface tension gradients, thermo-capillary convection (owing to temperature and concentration gradients) or from buoyancy (natural convection). In OFZ, convection in the liquid zone can have an effect on liquid zone length, the shape of the S/L interface or on zone stability. Forced convection can be introduced by rotations. Uniform (axisymmetric) heating is possible with slow rotation rates.

In the OFZ process there is a liquid-vapour (L-V) interface (often referred to as the liquid surface) with an imposed temperature gradient. The surface tension is a function of temperature. Thus, the temperature gradient will produce motion in the liquid (convection). The shape of the solid (S) depends on the L-V interface at the S-L-V junction.

The surface tension of hot liquid is lower compared with that of colder liquid. Thus, the system will attempt to lower its free energy via movement of the liquid from areas of low surface tension to areas of high surface tension. The resulting convection is referred to as surface driven flow, thermocapillary flow or Marangoni flow or Marangoni convection in temperature gradient (MCT). This is described by the thermal Marangoni number Ma(T) = PrM, where Pr is the Prandtl number (see below) and M is the surface tension parameter (see below). Gradients in composition will also produce such a flow that is referred to as Marangoni convection in concentration gradient (MCC). The buoyancy driven convection arises from interaction of density variations in the liquid (due to temperature variations) with gravity.

The Marangoni flow has an effect on transport phenomena (i.e., mass and heat transfer) during OFZ. The shape of the S/L interface and the distribution of solute (i.e., the development or not of chemical inhomogeneities) depend on the mass and heat transfer. The homogeneity of the solid is influenced by mass transfer and heat transfer, due to their effect on the shape of the S/L interface, and the shape of the solid and the stability of the floating zone are influenced by heat transfer. For Pr << 1, laminar flow has a small influence on the heat transfer.

Dimensionless numbers that are important for understanding OFZ processing include the surface tension parameter M, and the Prandtl Pr and Schmidt Sc numbers. The surface tension parameter is M = ρ(T_o_ − T_m_)(−∂γ_LV_/∂T)α/μ^2^ and is used to characterize thermocapillary flow in OFZ. In this equation ρ is the density of the liquid, α is the radius of the zone, μ is the liquid viscosity, T_o_ is the temperature of the liquid surface at the center of the zone (K) and T_m_ is the interface temperature taken to be the melting point (K). For elemental Nb the term − ρ(∂γ_LV_/∂T)/μ^2^ is ≈ 1380 (the data for Nb is from [52]), compared with M ≈ 14,000 for Si [52]. Thus, for elemental Nb and α = 5 mm (the diameter of the solid rods used for OFZ in this research was 10 mm) the value of M is 690, 345 and 34.5 for T_o_ − T_m_ equal to 1, 0.5 and 0.05 K, respectively. According to Chang and Wilcox [52] buoyancy driven convection in enclosed spaces is oscillatory for T_o_ − T_m_ above 0.1 K and turbulent above 2 K.

The Prandtl (Pr) number (=C_p_ μ/k) is the ratio of momentum diffusivity (μ/ρ = kinematic viscosity) to thermal diffusivity (k/ρC_P_) where C_P_ is the specific heat at constant pressure and k is the thermal conductivity. The Schmidt (Sc) number (=μ/ρD) is the ratio of momentum diffusivity to diffusion coefficient, where D is the diffusion coefficient. For elemental Nb the values are Pr ≈ 0.026 (compared with Pr ≈ 0.023 for Si and Pr ≈ 0.025 for Mo) and Sc ≈ 145 (compared with Sc ≈ 5 for Si) (the data for elemental Nb is from [52]). The Prandtl number Pr is important for the shape of the melt zone. When the power input increases, the shape of the S/L interface changes from convex towards the liquid to flat to concave [53].

Transport by convection becomes more significant compared with transport by conduction or diffusion as Pr or Sc increases. Liquid metals are low Pr number materials. Small value of the Prandtl number, Pr << 1, means the thermal diffusivity dominates and large Prandtl number, Pr >> 1, means the momentum diffusivity dominates. The Pr values for elemental Si and Nb indicate that the heat conduction is more significant compared with convection and that thermal diffusivity is dominant. Thus, if these elements were to be grown using OFZ, it is most likely that they will exhibit thermocapillary flow. The latter can introduce considerable inhomogeneities even at gentle flow. Thermocapillary flow instability in the liquid may appear before instability from the L-V interface sets in. The above could explain the chemical inhomogeneity between the bulk and edge of the OFZ bars grown at the three different growth rates that was observed in CM1-OFZ.

**Figure 8:**
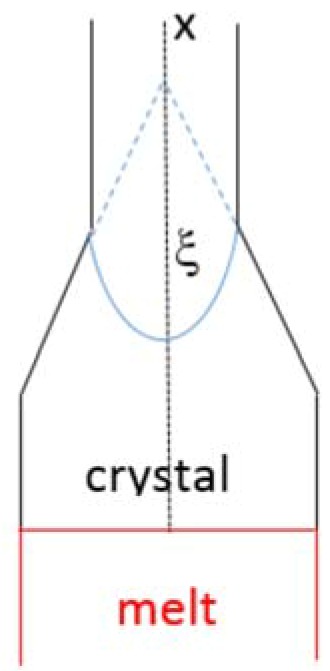
Schematic diagram showing the geometry considered by Louchev et al. [54].

Louchev et al. [54] used an 1D heat transfer problem to study the thermal stresses in the initial stages of crystal growth from the melt for the geometry shown in Figure 8, and considered the effect of change in cross sectional area with distance from the growth interface using the angle ξ. They proposed that the thermal stress is given by the equation σ_thermal_ ≈ αEw^2^(d^2^T/dx^2^) where α is the linear CTE, E is the Young’s modulus, w = 0.2R (R is the diameter of the crystal) and dT/dx is the temperature gradient in the crystal. Tensile stress (σ_thermal_ > 0) would arise from the heat dissipation from the lateral surface and compressive stress (σ_thermal_ < 0) from the radiation heat flux incoming to the crystal surface and from the geometry of the growing crystal (described using the angle ξ in Figure 8). For small ξ (<30°) the thermal stress is tensile. The temperature gradient in the solid (dT/dx)_S_ decreases with increasing ξ and the highest temperature gradient occurs for ξ = 0. During the OFZ processing of the alloy CM1 and at each growth rate, thermal stresses could have resulted from small or large change(s) in cross sectional area that could have occurred for any of the reasons discussed above. These thermal stresses would have interacted with any stresses that had resulted from CTE anisotropy of the alloyed Nb_5_Si_3_ (note that the surfaces of CM1-OFZ bars were not absolutely smooth cylindrical surfaces, see results).

In OFZ processing the formation of constitutionally undercooled liquid and therefore the “breakdown” of the interface is most likely at the centre rather than at the periphery of the solidifying solid. At low M values, if the growth rate is not low there will be radial variations in solute concentration. At intermediate M values oscillations and formation of striations parallel to the S/L interface are likely.

**Figure 9:**
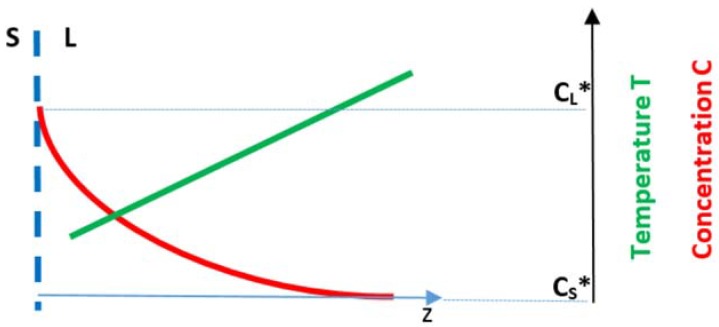
Schematic diagram showing concentration and temperature gradients ahead of an S/L interface.

Consider an alloy of composition C_o_ with solute having partition coefficient k < 1 and reducing the surface tension of the melt. A solute boundary layer is formed ahead of the S/L interface. The Figure 9 shows the position of the S/L interface at T = T* with the vertical dashed line (with the compositions of the solid and liquid at this temperature as C_S_* and C_L_*, respectively, which can be read on the axis on the right hand side), the solute rich boundary layer ahead of the interface (red curve) and the temperature gradient in the melt (green line). Assume that the alloy is processed using OFZ and allow for oscillations of temperature in the liquid.

According to Schwabe et al., the opposite forces, f_i_, acting on the S/L interface due to the temperature and concentration gradients, respectively will be fz(T) = (∂γ/∂T)(∂T/∂z) and fz(C) = (∂γ/∂C)(∂C/∂z), where γ is surface tension [55]. Consider fz(T) > fz(C). The resultant force means that phenomena will be driven by Marangoni convection in temperature gradient. This MCT will carry hot liquid to the interface and will support the heat transfer to the interface from the liquid ahead of the S/L interface. If there were to be an increase in V_S/L_ the solute profile in the boundary layer would change and the fz(C) will increase, which means that the resulting force on the interface will be reduced, i.e., the MCT will be reduced and this will result in an increase in V_S/L_. If instead there were to be a decrease in V_S/L_, the solute profile in the boundary layer would change and the fz(C) will decrease, which means that the resulting force on the interface will be increased, i.e., the MCT will be increased, meaning the MCT carries more hot liquid to the interface making it more morphologically stable. This simple argument shows how growth instabilities could have resulted from MCT and MCC and also provides another “mechanism” for counter-balancing the stresses that arise from the CTE anisotropy of alloyed Nb_5_Si_3_.

In floating zone melting the temperature gradient in the liquid (dT/dx)_L_ is linked with that in the solid (dT/dx)_S_ via the equation k_S_(dT/dx)_S_ = k_L_(dT/dx)_L_ − V_S/L_∆Hρ_S_ where k_i_ (i = S, L) is thermal conductivity, ∆H is enthalpy and ρ_S_ is the density of the solid. For growth at a constant rate, a decrease in (dT/dx)_S_ is linked with a decrease in (dT/dx)_L_ and thus would affect the conditions for forming constitutionally undercooled liquid ahead of the S/L interface and therefore the morphological stability of the latter. As (dT/dx)_S_ decreases with increasing ξ (see Figure 8) a change in cross section of the crystal (i.e., change in ξ), which results to compressive thermal stress in the crystal (see above), will also reduce (dT/dx)_L_ (=G_L_ in Figure 9) and thus encourage the onset of constitutional undercooling [54]. This simple argument provides another “mechanism” for counter-balancing the stresses that arise from the CTE anisotropy of alloyed Nb_5_Si_3_.

### 4.2. Heat-Treated Alloy

After the heat treatment the phases present in the microstructures of CM1-10g, CM1-600g, CM1-6mm, CM1-8mm and CM1-OFZ were the Nb_ss_ and αNb_5_Si_3_ and HfO_2_. The former two phases (and the absence of Nb_3_Si) is in accordance with the ternary Nb-Si-Mo and Nb-Si-W phase equilibria at 1700 °C [40,41].

With the exception of CM1-600g, in which there were Nb_ss_ grains with no Si and grains where the solubility of Si in the Nb_ss_ was low and at similar concentration to that reported for the Nb_ss_ in heat-treated Nb-silicide-based alloys [6,28,33], in all other forms of the alloy CM1 the Nb_ss_ was free of Si (i.e., Nb with no Si, see [34]), in agreement with other research for arc melted Nb-silicide alloys with refractory metal additions [34,35].

Precipitation of second phase with contrast similar to that of Nb_ss_ has been reported by our group in αNb_5_Si_3_ in the heat-treated Nb-24Ti-18Si-5Al [56] and Nb-18Si-5Al-5Cr-5Mo alloys [43] and in αNb_5_Si_3_ and/or βNb_5_Si_3_ in the heat-treated Nb-24Ti-18Si-5Al-5Cr, Nb-24Ti-18Si-6Ta-5Al-5Cr, Nb-24Ti-18Si-8Cr-4Al, Nb-24Ti-18Si-6Ta-8Cr-4Al [18,56], Nb-24Ti-18Si-5Al-5Cr-5Mo, Nb-24Ti-18Si-5Al-5Cr-2Mo [43], Nb-24Ti-18Si-5Al-5Cr-5Hf-5Sn-2Mo [57] and Nb-18Si-5Al-5Ge and Nb-24Ti-18Si-5Al-5Ge [33] alloys. Precipitation of Nb_ss_ in αNb_5_Si_3_ in Nb-20Ti-18Si-4Hf-5Cr-3Al-1.5Sn also has been reported by Cheng et al. [58]. In the latter alloy the orientation relationship (222)_Nbss_ // (002)_αNb5Si3_, [1$\bar{1}$0]_Nbss_ // [1$\bar{1}$0]_αNb5Si3_ was reported, and the interface between Nb_ss_ and αNb_5_Si_3_ was found to be enriched in Hf.

For the heat-treated alloy Nb-24Ti-18Si-5Al, Zelenitsas and Tsakiropoulos [18] suggested that the Nb_ss_ precipitates were the product of the βNb_5_Si_3_ → αNb_5_Si_3_ + Nb_ss_ phase transformation. The data in [18,56] showed that in the heat-treated Nb-24Ti-18Si-5Al, Nb-24Ti-18Si-6Ta-5Al-5Cr, Nb-24Ti-18Si-8Cr-4Al and Nb-24Ti-18Si-6Ta-8Cr-4Al alloys the Nb_ss_ precipitates did not form in Ti rich areas in the αNb_5_Si_3_ and/or βNb_5_Si_3_. The data from our research group [18,33,43,57] and Cheng et al. [58] would suggest that precipitation of Nb_ss_ in βNb_5_Si_3_ and/or αNb_5_Si_3_ is a more general phenomenon in Nb-silicide-based alloys and that many solutes contribute to this precipitation.

Precipitation of Nb_ss_ was observed in the cast alloy CM1 (a) in subgrained αNb_5_Si_3_ in CM1-600g, and (b) in the non-subgrained αNb_5_Si_3_ in CM1-OFZ grown at 150 mm/h, 60 mm/h and 12 mm/h. Precipitation of Nb_ss_ was observed in the heat-treated CM1 (c) in all the subgrained αNb_5_Si_3_ in the heat-treated CM1-10g, CM1-6mm, CM1-8mm, CM1-600g, and CM1-OFZ (for all three growth rates), see Table S1 in supplemental data. 

The formation of subgrains in the αNb_5_Si_3_ in the heat-treated CM1-OFZ bars grown at the three different growth rates was attributed to the strain energy reduction via “recovery” phenomena, and the strain energy was attributed to the anisotropy of CTE due to the partitioning of solutes.

Figure 5g shows the microstructure of an αNb_5_Si_3_ grain with fine precipitates. The subgrain boundary exhibits dark contrast but the bulk of the subgrains exhibits lighter contrast, meaning the latter must be richer in high atomic number elements (Hf, W). In this alloy the Nb_ss_ in CM1-10g was also rich in W. Please note that near the subgrain boundary some precipitates are larger in size. Overall the Nb_ss_ precipitates were of nanometer size and the spacing between them was also in the nanometer scale. Precipitates with high aspect ratio together with fine spherical precipitates can be seen in Figure 2d and Figure 6b. In Figure 2d and Figure 6 it should be noted that there is a precipitate free area (zone) near the interface of Nb_ss_ and αNb_5_Si_3_.

In the alloy CM1, in the βNb_5_Si_3_ the high Ti concentration was associated with low Mo (and W) concentrations (the Hf and W concentrations were very low, practically zero), and Nb_ss_ precipitates formed in the large silicide grains in areas where X-ray maps (Figure S5 in supplemental data) indicated low Ti and high Mo content, and there was a “band” of Ti rich and Mo poor silicide that was precipitate free. Such bands were formed next to the Nb_ss_ surrounding the Nb_5_Si_3_. In the Nb_ss_ the high Ti concentration was associated with low Mo and W content, there were Ti rich and Ti poor areas in the Nb_ss_ after the heat treatment, and the W concentration in the Nb_ss_ also varied significantly. Given that no precipitates were observed in the Ti rich (and Mo poor) areas (bands) formed at the interface between Nb_ss_ and Nb_5_Si_3_ (which according to the literature [18,56] are the areas where Ti segregates during the solidification of Nb-silicide-based alloys), the precipitation of Nb_ss_ occurred in the Ti poor and Mo rich areas in the bulk of the silicide, which is in agreement with previous research (see above) and the role of Mo in the precipitation of Nb_ss_ according to the work of Sekido et al. [42]. The Figure 2d, Figure 4f, Figure 5g would suggest that there was some orientation relationship of the Nb_ss_ precipitates in the silicide grain.

## 5. Conclusions

In this work a Nb-silicide-based alloy of near eutectic composition Nb-21.1Si-8.3Ti-5.4Mo-4W-0.7Hf (alloy CM1) was studied in the cast and heat-treated conditions. The alloy was produced in the form of buttons and bars using three different methods, namely arc-melting, arc-melting and suction casting, and OFZ. In the former two cases the alloy solidified in water-cooled copper crucibles and buttons of different size (weight) were produced.

The type of Nb_5_Si_3_ observed in the cast microstructures of the alloy CM1 depended on the solidification conditions.

The primary phase in the alloy CM1 was the βNb_5_Si_3_.

The results confirmed that the transformation βNb_5_Si_3_ → αNb_5_Si_3_ had occurred in large size button and the OFZ bars of the alloy CM1.

The transformation βNb_5_Si_3_ → αNb_5_Si_3_ was accompanied by subgrain formation in the silicide and the precipitation of a second phase with same contrast as the Nb_ss_.

The partitioning of solutes and in particular of Mo and Ti was key to this phase transformation.

Subgrain formation was not necessary for precipitation of Nb_ss_ in αNb_5_Si_3_. The partitioning of solutes was essential for this precipitation.

## Figures and Tables

**Table 1:** Summary of data for type(s) of silicide(s) in alloy CM1.

Condition	Button (Weight—g)		Suction-Cast Bar (Diameter—mm)		OFZ (Growth Rate—mm/h)		
	10	600	6	8	150	60	12
As cast							
αNb_5_Si_3_	-	X *	-	-	X *	X *	X *
βNb_5_Si_3_	X	-	X	X	-	-	-
Nb_3_Si ** Nb_3_Si → Nb_ss_ + αNb_5_Si_3_	-	-	-	X	-	-	-
Heat treated							
αNb_5_Si_3_	X *	X *	X *	X *	X *	X *	X *
βNb_5_Si_3_	-	-	-	-	-	-	-

* Precipitates exhibiting same contrast as Nb_ss_ in silicide grains; ** Observed only in one part of CM1-8mm where solidification had occurred at high cooling rate.

**Table 2:** Analysis data (at.%) of cast CM1-OFZ.

Element	Large Area		Lamellar Microstructure		Silicide		Solid Solution	
	Bulk	Edge	Bulk	Edge	Bulk	Edge	Bulk	Edge
**150 mm/h**								
**Nb**	60.2 ± 0.34	58.7 ± 0.47	60.6 ± 0.71	61.4 ± 0.53	52.6 ±0.92	53.3 ± 0.26	70.4 ± 0.90	69.8 ± 0.66
	59.7–60.6	58.1–59.3	59.9–61.9	60.9–62.2	50.9–53.5	52.9–53.6	69.1–71.8	68.7–70.4
**Ti**	9.2 ± 0.39	8.6 ± 0.77	8.3 ± 1.05	7.2 ± 0.63	7.6 ± 1.19	7.0 ± 0.08	10.7 ± 1.91	6.6 ± 0.74
	8.8–9.7	7.4–9.5	7.0–9.7	6.7–8.4	6.7–9.7	7.0–7.2	8.5–13.5	5.5–7.4
**Si**	20.6 ± 0.46	23.7 ± 1.31	20.3 ± 0.62	18.7 ± 0.96	37.4 ± 0.76	37.3 ± 0.66	1.8 ± 0.44	1.6 ± 0.45
	20.2–21.4	22.1–25.2	19.0–20.9	17.3–19.9	37.0–38.6	36.5–38.1	1.0–2.2	1.2–2.4
**Mo**	6.3 ± 0.25	5.7 ± 0.38	6.8 ± 0.35	7.4 ± 0.42	1.7 ± 0.17	2.0 ± 0.32	12.0 ± 0.63	13.1 ± 0.41
	6.0–6.6	5.2–6.2	6.2–7.3	6.7–7.9	1.5–2.0	1.6–2.6	11.3–13.0	12.8–13.8
**Hf**	1.2 ± 0.16	1.0 ± 0.13	1.0 ± 0.20	0.9	0.5	0.3	0.7	0.6
	1.0–1.3	0.8–1.2	0.8–1.4	0.7–1.1	0.4–0.6	0.2–0.4	0.5–0.8	0.4–0.8
**W**	2.5 ± 0.16	2.4 ± 0.22	3.1 ± 0.65	4.3 ± 0.52	0.1	0.1	4.5 ± 1.19	8.3 ± 0.51
	2.3–2.7	2.0–2.7	2.2–4.0	3.4–4.7	0.0–0.1	0.1–0.1	3.2–6.5	7.7–9.0
**60 mm/h**								
**Nb**	61.2 ± 0.41	59.6 ± 0.21	61.1 ± 0.32	61.4 ± 0.68	53.3 ± 0.32	53.3 ± 0.39	71.0 ± 0.61	70.1 ± 0.53
	60.7–61.8	59.4–59.9	60.6–61.6	60.6–62.7	53.1–53.9	52.8–53.9	70.0–71.9	69.4–70.9
**Ti**	8.9 ± 0.68	7.7 ± 0.40	8.2 ± 0.61	7.3 ± 0.34	6.9 ± 0.13	6.9 ± 0.13	8.8 ± 0.46	7.3 ± 1.00
	8.1–9.6	7.2–8.3	7.1–9.0	6.9–7.9	6.8–7.1	6.7–7.1	7.9–9.2	5.8–8.8
**Si**	19.6 ± 0.88	22.5 ± 0.6	20.6 ± 0.88	19.5 ± 1.17	37.8 ± 0.55	37.5 ± 0.97	1.8 ± 0.64	1.7 ± 0.65
	18.5–21.0	21.6–23.3	19.4–21.4	17.4–20.8	36.9–38.5	36.0–39.0	0.9–2.8	1.0–2.7
**Mo**	6.4 ± 0.21	6.1 ± 0.23	6.4 ± 0.35	7.0 ± 0.40	1.6 ± 0.29	1.8 ± 0.34	12.1 ± 0.74	12.9 ± 0.62
	6.1–6.7	5.8–6.4	5.6–6.8	6.5–7.6	1.1–1.9	1.4–2.4	11.1–13.0	12.0–13.5
**Hf**	1.2 ± 0.2	1.0 ± 0.12	0.8	0.9	0.3	0.4	0.9	0.6
	0.9–1.4	0.9–1.2	0.8–0.9	0.7–1.2	0.2–0.4	0.3–0.5	0.6–1.0	0.4–0.8
**W**	2.8 ± 0.35	3.1 ± 0.21	2.9 ± 0.11	3.9 ± 0.56	0.1	0.1	5.4 ± 0.67	7.5 ± 137
	2.2–3.2	2.9–3.4	2.8–3.1	2.8–4.6	0.1–0.1	0.0–0.2	4.3–6.3	5.9–9.4
**12 mm/h**								
**Nb**	59.7 ± 0.58	58.7 ± 0.26	60.3 ± 0.20	60.0 ± 0.82	52.8 ± 0.14	52.9 ± 0.22	70.1 ± 0.52	68.7 ± 0.96
	58.6–60.4	58.3–59.0	60.1–60.7	58.7–61.1	52.6–53.0	52.4–53.0	69.1–70.5	67.8–70.1
**Ti**	9.1 ± 1.27	7.3 ± 0.37	8.0 ± 1.41	7.6 ± 0.56	7.5 ± 0.31	7.3 ± 0.30	8.1 ± 0.87	6.7 ± 0.80
	7.6–11.2	6.8–7.8	6.7–10.5	6.9–8.4	7.2–8.1	6.8–7.7	7.2–9.3	5.8–7.6
**Si**	20.8 ± 1.69	21.0 ± 0.89	20.1 ± 0.63	19.6 ± 1.15	37.3 ± 0.56	37.1 ± 0.67	2.6 ± 0.49	1.7 ± 0.53
	18.7–23.2	20.1–22.4	19.0–20.8	18.8–21.9	36.6–38.0	36.2–38.2	1.7–3.3	0.7–2.1
**Mo**	6.2 ± 0.75	7.9 ± 0.50	6.8 ± 0.37	7.3 ± 0.39	1.9 ± 0.32	2.2 ± 0.26	11.7 ± 0.70	12.6 ± 0.69
	4.9–7.2	6.9 – 8.1	6.2–7.3	6.8–7.8	1.3–2.2	1.9–2.5	10.7–12.6	11.9–13.1
**Hf**	1.1 ± 0.12	0.2 ± 0.17	0.9 ± 0.10	0.9 ± 0.16	0.4	0.3	0.7	0.7
	1.0–1.3	0.0–0.4	0.8–1.1	0.7–1.1	0.3–0.5	0.2–0.5	0.5–0.8	0.5–1.0
**W**	3.1 ± 0.43	4.9 ± 0.12	3.8 ± 1.10	4.5 ± 0.39	0.1	0.1	6.7 ± 1.06	9.6 ± 1.59
	2.4–3.6	4.7–5.0	2.2–5.1	3.9–4.6	0.1–0.1	0.1–0.2	5.2–8.0	7.4–11.6

## Supplementary Materials

The following are available online at http://www.mdpi.com/1996-1944/11/6/967/s1, Figure S1: X-ray diffractograms of cast and heat treated CM1-10g and CM1-600g, Figure S2: X-ray diffractograms of cast and heat treated CM1-6mm and CM1-8 mm bars, Figure S3: X-ray diffractograms of cast CM1-OFZ grown at growth rates of 12, 60 and 150 mm/h, Figure S4: Relationships between W and Ti concentrations in the Nbss (a) in as-cast CM1-10g and (b) CM1-OFZ where data for all three growth rates is included, Figure S5: Backscattered electron image of a silicide in as-cast CM1-OFZ grown at 150 mm/hr and X-ray maps of the elements in the alloy, Figure S6: X ray diffractograms of heat treated CM1-OFZ grown at 12, 60 and 150 mm/h, Table S1: Summary of microstructures in arc melted, suction cast and OFZ alloy CM1.
