# Supplementary material for: Type of Primary Nb5Si3 and Precipitation of Nbss in αNb5Si3 in a Nb-8.3Ti-21.1Si-5.4Mo-4W-0.7Hf (at.%) Near Eutectic Nb-Silicide-Based Alloy
Source: Materials (Basel). 2018 Jun 7;11(6):967. doi: 10.3390/ma11060967 (PMC6024954; doi:10.3390/ma11060967)
Supplement: Supplementary file 1 [file materials-11-00967-s001.pdf]

Table S1: Summary of microstructures in arc melted, suction cast and OFZ alloy CM1

Alloy CM1-	Condition	
	As cast	Heat treated
10 g	Nb_{ss}, βNb_5Si_3<br>Nb_{ss} + βNb_5Si_3 eutectic	Nb_{ss}, αNb_5Si_3<br>Nb_{ss} + αNb_5Si_3 lamellar microstructure<br>subgrains in Nb_5Si_3 and fine precipitates
6mm	Nb_{ss}, βNb_5Si_3<br>Nb_{ss} + βNb_5Si_3 eutectic	Nb_{ss}, αNb_5Si_3<br>Nb_{ss} + αNb_5Si_3 lamellar microstructure<br>subgrains in Nb_5Si_3 and fine precipitates
8mm	Nb_{ss}, Nb_3Si, βNb_5Si_3<br>Nb_{ss} + βNb_5Si_3 eutectic<br>$Nb_3Si \rightarrow Nb_{ss} + \alpha Nb_5Si_3$	Nb_{ss}, αNb_5Si_3<br>Nb_{ss} + αNb_5Si_3 lamellar microstructure<br>subgrains in Nb_5Si_3 and fine precipitates
600 g	Nb_{ss}, αNb_5Si_3<br>Nb_{ss} + αNb_5Si_3 lamellar microstructure<br>subgrains in Nb_5Si_3 and fine precipitates	Nb_{ss}, αNb_5Si_3<br>Nb_{ss} + αNb_5Si_3 lamellar microstructure<br>subgrains in Nb_5Si_3 and precipitates
OFZ All growth rates	Nb_{ss}, αNb_5Si_3<br>Nb_{ss} + αNb_5Si_3 lamellar microstructure<br>Fine precipitates in αNb_5Si_3	Nb_{ss}, αNb_5Si_3<br>Nb_{ss} + αNb_5Si_3 lamellar microstructure<br>subgrains in Nb_5Si_3 and fine precipitates

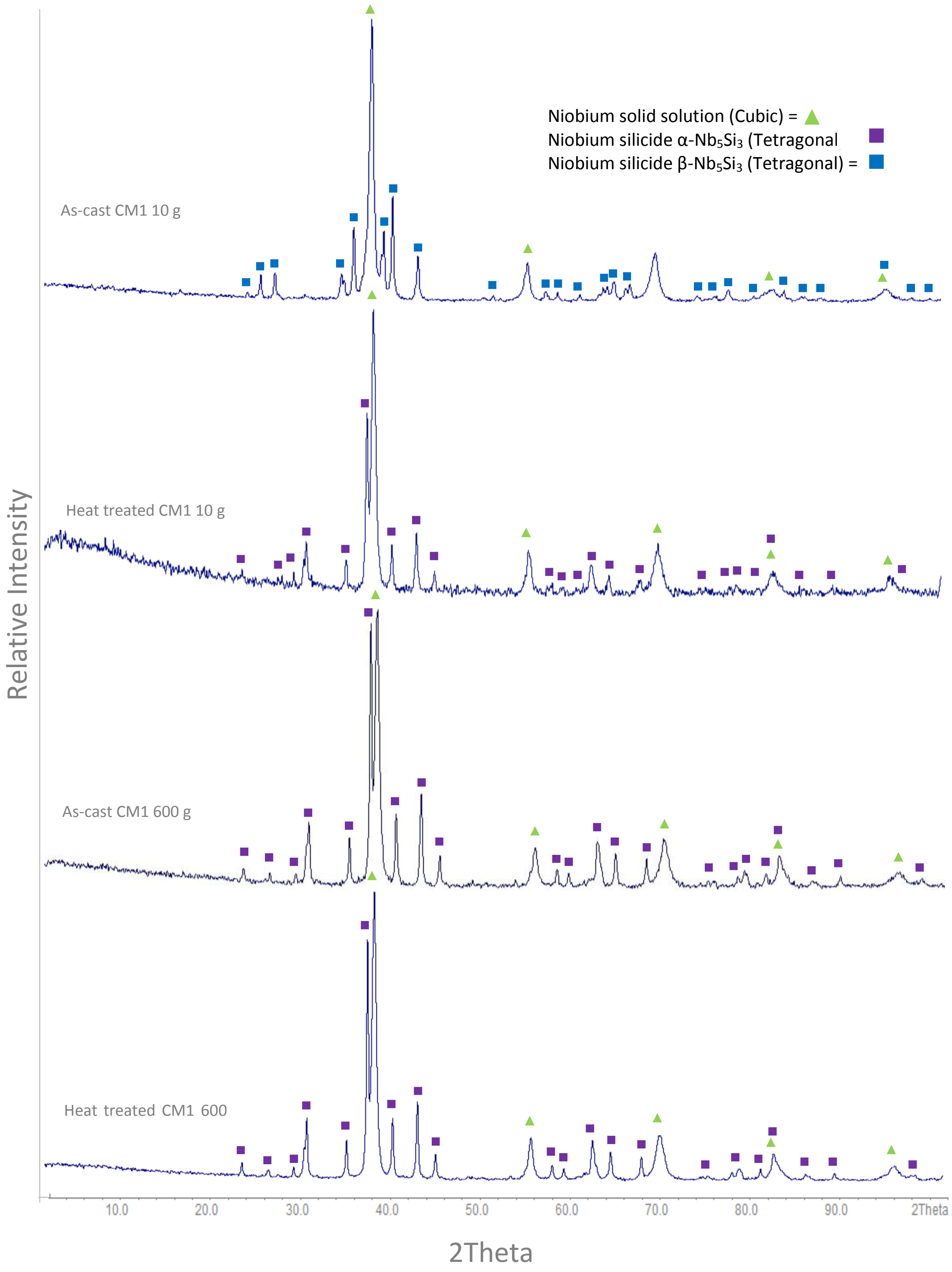

Figure S1: X-ray diffractograms of cast and heat treated CM1-10g and CM1-600g

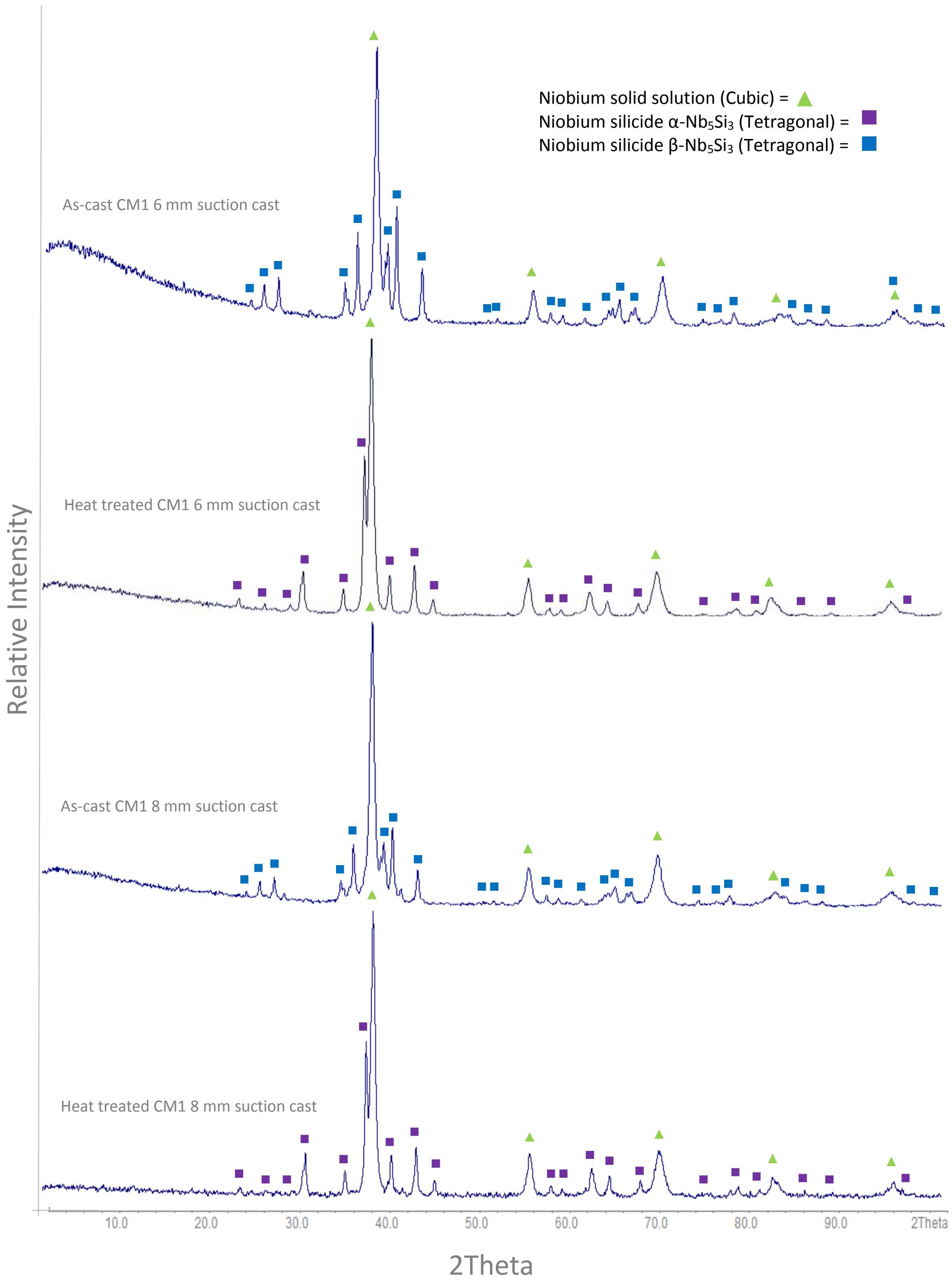

Figure S2: X-ray diffractograms of cast and heat treated CM1-6mm and CM1-8 mm bars

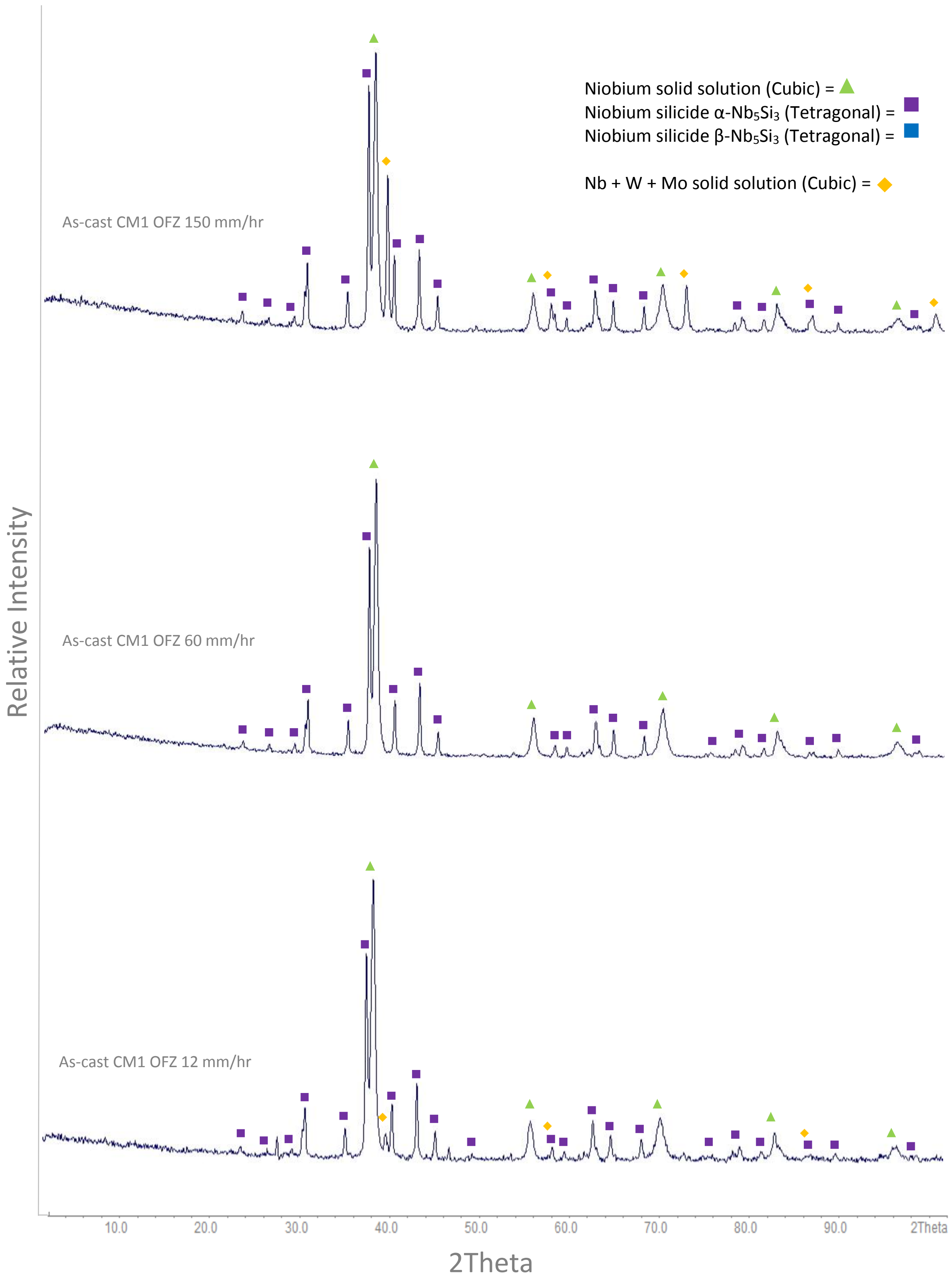

Figure S3: X-ray diffractograms of cast CM1-OFZ grown at growth rates of 12, 60 and 150 mm/h.

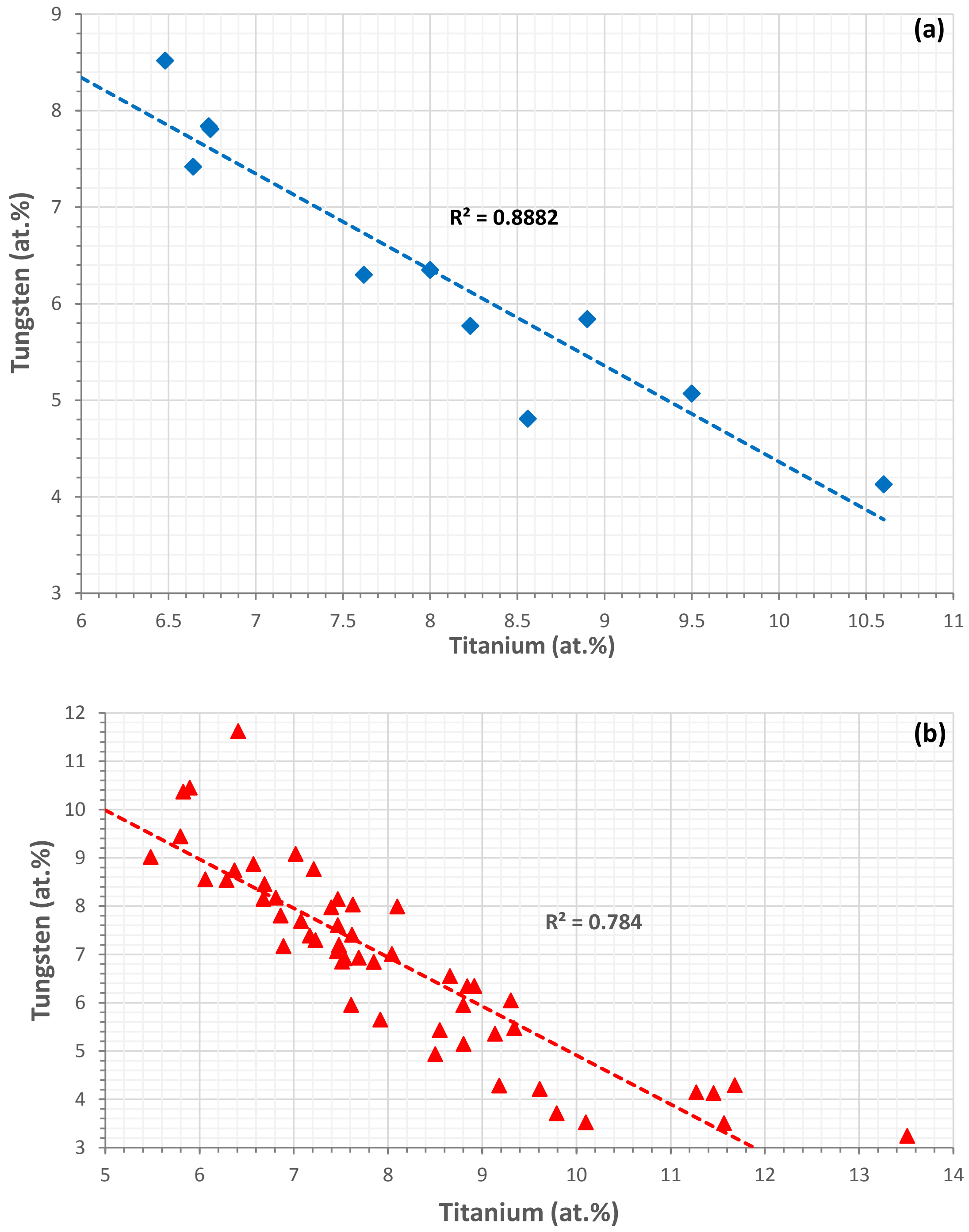

Figure S4: Relationships between W and Ti concentrations in the Nb_{ss} (a) in as-cast CM1-10g and (b) CM1-OFZ where data for all three growth rates is included.

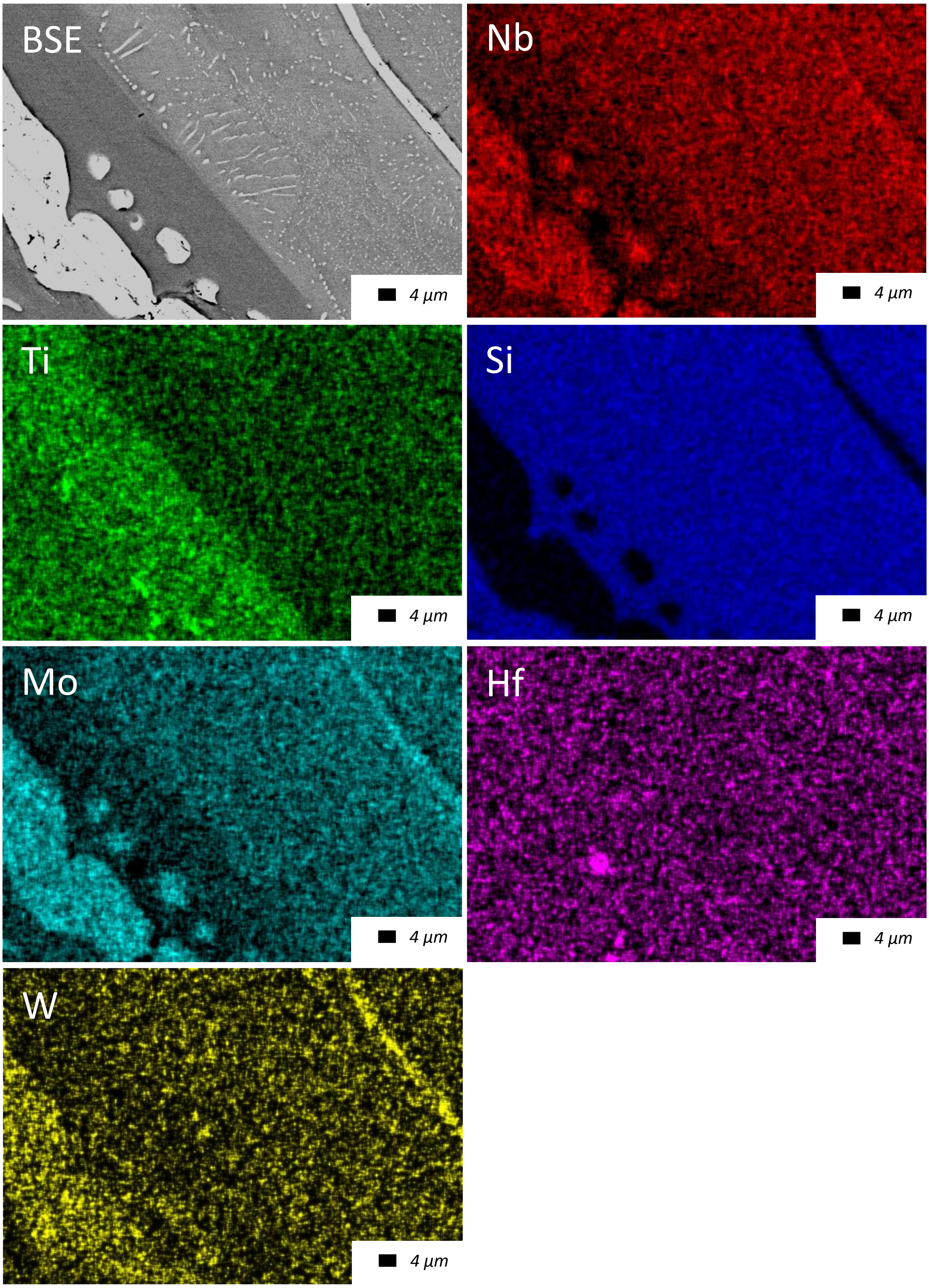

Figure S5: Backscattered electron image of a silicide in as-cast CM1-OFZ grown at 150 mm/hr and X-ray maps of the elements in the alloy.

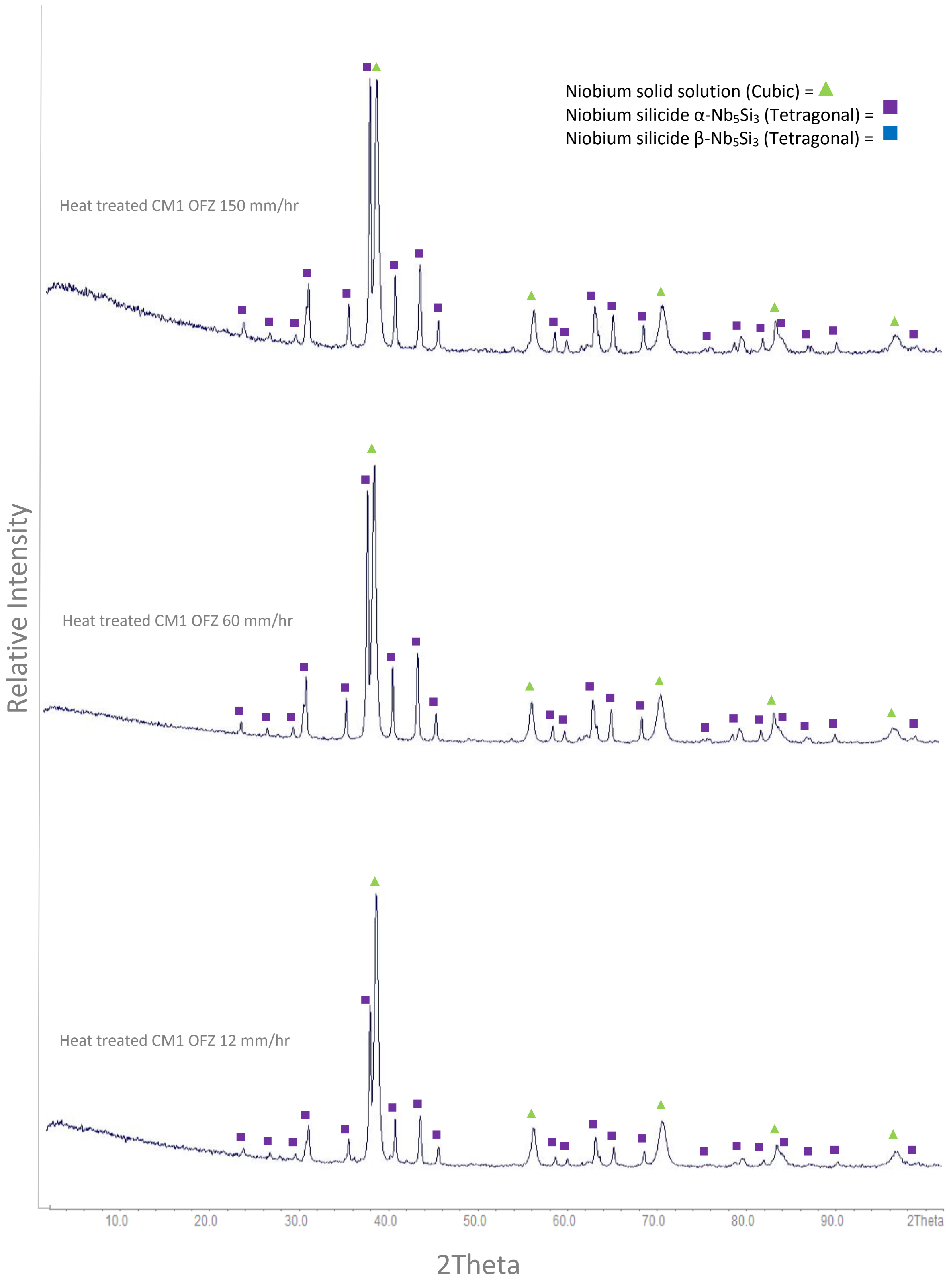

Figure S6: X ray diffractograms of heat treated CM1-OFZ grown at 12, 60 and 150 mm/h
